# Dichotomous SMAD2/3 regulation and selective antihypertrophic activity of heparin during in vitro chondrogenesis of mesenchymal stromal cells

**DOI:** 10.1186/s11658-026-00899-8

**Published:** 2026-03-17

**Authors:** Sven Schmidt, Safak Chasan, Helen F. Dietmar, Felicia A. M. Klampfleuthner, Eliane Hesse, Tilman Walker, Uwe Freudenberg, Wiltrud Richter, Solvig Diederichs

**Affiliations:** 1 Experimental Orthopaedics, Research Centre for Molecular and Regenerative Orthopaedics, Department for Orthopaedics, Heidelberg University Hospital, Schlierbacher Landstraße 200a, 69118 Heidelberg, Germany; 2 Department for Orthopaedics, Heidelberg University Hospital, Heidelberg, Germany; 3 Leibniz Institute of Polymer Research Dresden (IPF), Max Bergmann Centre of Biomaterials Dresden (MBC), Centre for Regenerative Therapies Dresden (CRTD), Dresden University of Technology, Dresden, Germany

**Keywords:** Heparin, Heparan sulfate, Endochondral development, Chondrocyte hypertrophy, TGF-β, WNT/β-catenin, SMAD, AKT, Prostaglandin, Stem cells

## Abstract

**Background:**

Endochondral instead of chondral differentiation hinders mesenchymal stromal cell (MSC) application for clinical cartilage regeneration. We previously showed that heparin–polyethylene glycol (PEG) hydrogels loaded with transforming growth factor TGF-β instructed stable chondral MSC development in vivo. Here, we assessed this approach in vitro, utilizing heparin–PEG hydrogels or the pellet culture system with soluble heparin supplementation of chondrogenic medium.

**Methods:**

Human MSCs were cultured in heparin–PEG hydrogels (22.4 mg/mL crosslinked heparin, 120 ng TGF-β1) or as pellet cultures treated with soluble heparin (0, 10, 100, 700 μg/mL) in TGF-β1-containing (10 ng/mL) chondrogenic medium. Chondral and endochondral signaling (1–3 h, 4 weeks) and cartilage matrix formation (4 weeks) were analyzed using western blot, histology, quantitative polymerase chain reaction (qPCR), enzyme-linked immunosorbent assay (ELISA), and enzyme activity.

**Results:**

Unlike in vivo, human MSCs differentiated in heparin–PEG hydrogels into type X collagen and alkaline phosphatase-positive hypertrophic chondrocytes in vitro. Interestingly, treating MSC pellets with soluble heparin (10–700 µg/mL) revealed reduced TGF-β-SMAD3 but not SMAD2 activation at unaffected type II collagen and proteoglycan/DNA levels. We propose that the stimulation of the insulin-AKT pathway by heparin aided in maintaining SMAD2 activation, which apparently plays a more prominent role than SMAD3 for MSC chondrogenesis. Heparin treatment inhibited the pro-hypertrophic WNT/β-catenin pathway in vitro but insufficiently silenced TGF-β-SMAD1/5/9 activation and unfortunately reduced antihypertrophic prostaglandin PGE2 levels. Ultimately, treatment with 10 µg/mL heparin reduced the upregulation of several hypertrophy markers (*MEF2C*, *IHH*, *IBSP* messenger RNAs [mRNAs], alkaline phosphatase activity) below control levels, but type X collagen remained unresponsive. Thus, soluble heparin treatment was similarly selective and effective as previous antihypertrophic interventions (PTHrP pulses, WNT inhibition), while offering technical simplicity, reduced cost, and solvent-free formulation.

**Conclusions:**

Taken together, heparin-TGF-β showed a novel dichotomous SMAD2/3 inhibition at maintained chondrogenic differentiation and context-dependent lineage-instructive properties—permitting endochondral commitment in vitro but chondral development in vivo. Thus, environmental contributions are mandatory to allow heparin–PEG-guided chondral versus endochondral lineage commitment of MSCs in vivo, potentially involving SMAD1/5/9 suppressors and PGE2 sources.

**Graphical abstract:**

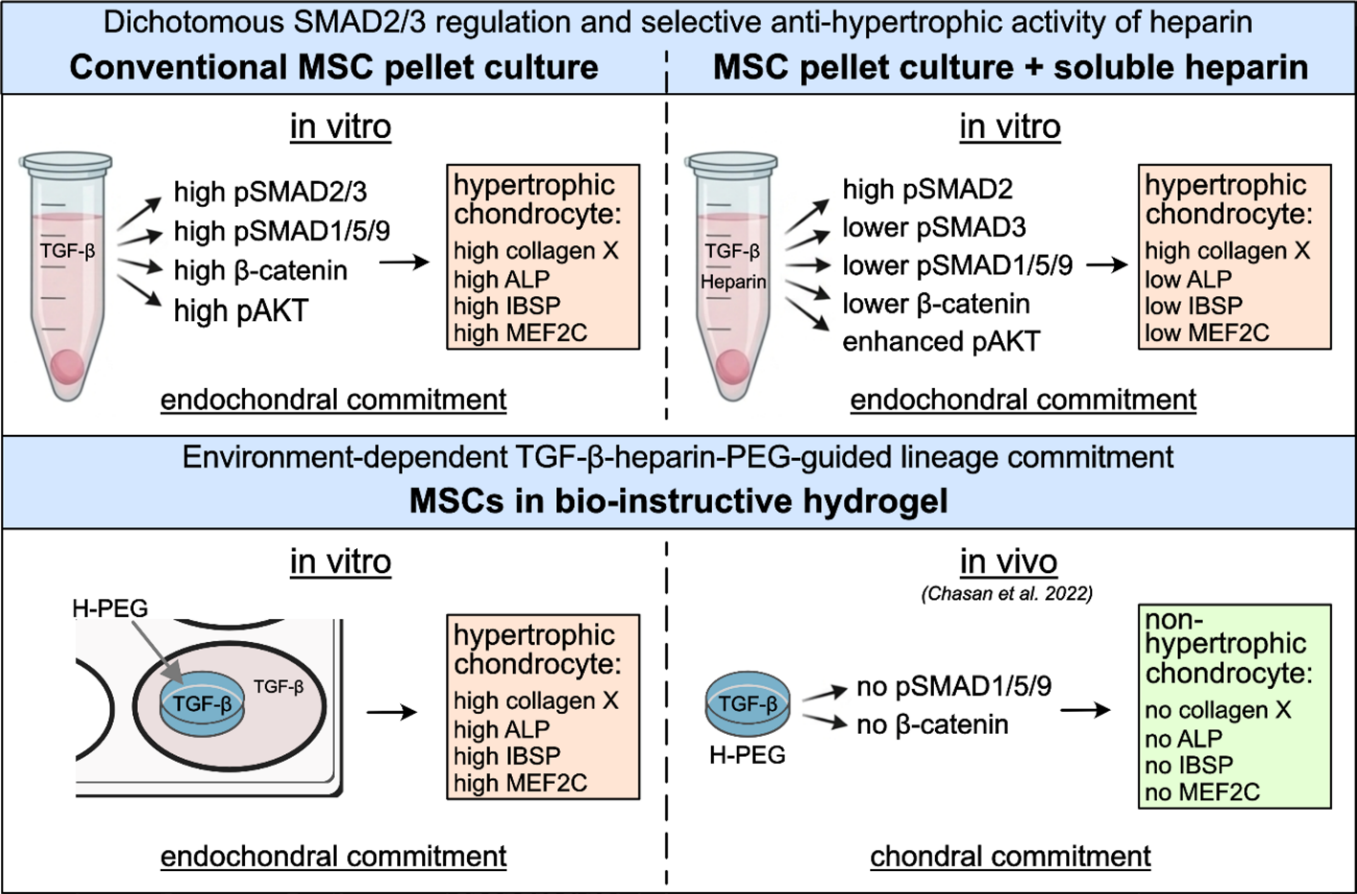

**Supplementary Information:**

The online version contains supplementary material available at 10.1186/s11658-026-00899-8.

## Introduction

Mesenchymal stromal cells (MSCs) are a promising autologous chondrocyte source for regenerating cartilage tissues. Cartilage regenerative strategies often involve in vitro chondrogenic MSC predifferentiation to avoid dependence on uncontrollable local in vivo signals for the directed differentiation into chondrocytes and the in situ formation of neocartilage. Application of predifferentiated rather than nondifferentiated MSC-based cartilage grafts can significantly enhance extracellular matrix deposition and improve repair quality [1, 2].

Standard MSC in vitro chondrogenesis conditions employ the essential chondro-inductive transforming growth factor TGF-β, which activates the pro-chondrogenic small mother against decapentaplegic (SMAD)2 and SMAD3 [3]; and insulin/AKT signaling cooperates in activating SMAD2 [4]. SMAD2/3 induce the chondrogenic master transcription factor SRY-box transcription factor (SOX)9, initiating the secretion of the hallmark extracellular cartilage matrix proteins type II collagen and the proteoglycan aggrecan [3].

Yet, under these conditions, MSCs unavoidably commit to the endochondral instead of the desired chondral cell lineage in vitro and phenocopy the endochondral bone development and growth of the skeleton [3]. Cells develop into hypertrophic chondrocytes that additionally release proteins associated with matrix mineralization and bone formation, such as type X collagen, alkaline phosphatase (ALP), and integrin-binding sialoprotein (IBSP) [5–7]. In vivo, the resulting hypertrophic neocartilage is prone to mineralize and to remodel into bone tissue [5], a highly undesired outcome for cartilage regeneration.

The current understanding of the signals driving the hypertrophic MSC misdifferentiation involves a complicated interplay between TGF-β-SMAD1/5/9 activity, cell-autonomous wingless-int (WNT) signaling, and dysregulation of the feedback loop between parathyroid hormone-related protein (PTHrP) and Indian Hedgehog (IHH) [8–10]. At the initiation of chondrogenesis, TGF-β noncanonically activates SMAD1/5/9 [11], which is associated with bone morphogenetic protein signaling, which promotes chondrocyte hypertrophy in the growth plate [12]. Additionally, TGF-β stimulates β-catenin accumulation in MSCs undergoing chondrogenesis [13], the mediator of canonical WNT signaling. Knockout and inhibitor studies demonstrated that WNT activity drives chondrocyte hypertrophy in both the growth plate and MSC chondrogenesis [8, 14, 15]. Furthermore, once PTHrP expression is downregulated during advancing MSC chondrogenesis, WNT signaling contributes to IHH upregulation [8], a prominent driver of endochondral ossification in the growth plate [16].

In line with these insights, WNT inhibition and pulsed PTHrP treatment are currently the most effective antihypertrophic interventions during MSC chondrogenesis [8, 15, 17]. Along with a strong reduction of ALP protein levels by 80% or more, WNT inhibition and PTHrP pulses reduce the expression of many hypertrophy markers, including *IBSP* and *IHH* mRNAs. However, type X collagen deposition remained unaffected, and the generated neocartilage mineralized and was remodeled into bone tissue after subcutaneous implantation, indicating a still insufficient instruction and stabilization of MSC commitment into the chondral cell lineage.

A major success in directing MSCs into the desired chondral lineage was to harness the lineage-determining capacity of sulfated glycosaminoglycans (sGAGs) of the heparan sulfate family on stem and progenitor cell development [18]. The unique structural features of heparan sulfates, most importantly their sulfation, enable them to interact with growth factors, morphogens, and cell surface receptors. Heparin is a highly charged heparan sulfate analogue and has a strong affinity to TGF-β [19, 20]. By utilizing heparin to immobilize TGF-β in a novel biodegradable poly(ethylene glycol) (PEG)-based hydrogel, we could instruct MSC commitment in vivo into the chondral lineage for the first time [18]. Allowing long-term retention of TGF-β and silencing WNT/β-catenin and SMAD1/5/9 pathways, heparin facilitated the formation of permanent neocartilage with high levels of type II collagen, while remaining remarkably free of type X collagen and exhibiting long-term resistance to calcification. However, this was achieved in the undefined environment of a subcutaneous mouse model, and it remains unclear whether similar success can be replicated under the defined conditions of in vitro chondrogenesis.

In our previous in vitro experiments with heparin–PEG hydrogels, MSC chondrogenesis resulted in mostly pericellular cartilage matrix deposition, significantly lower than that of articular chondrocytes [21]. Excessive TGF-β immobilization and sequestration in these hydrogels likely limited chondrogenic MSC differentiation, thus confounding the analysis of chondrocyte hypertrophy. By contrast, robust chondrogenic differentiation and homogeneous matrix deposition was achieved from goat MSCs or human fetal chondroprogenitor cells when supplying soluble TGF-β exogenously to heparin–PEG-gelatin or heparin–hyaluronan hydrogels, respectively [22, 23]. Levinson et al. reported positive type X collagen staining in their generated neocartilage, but a potential regulation by heparin remained unclear owing to a lack of heparin-free controls [23]. Importantly, our hydrogels incorporated over 20-fold more crosslinked heparin (2.24% versus 0.1% w/v) and may thus be more effective in suppressing type X collagen production.

Heparin has alternatively been supplied to chondrogenic cultures as soluble salt. Early studies using chick limb bud mesenchymal cells reported that low heparin concentrations (1–10 μg/mL) can stimulate proteoglycan synthesis, whereas higher concentrations (200 μg/mL) were detrimental [24, 25]. While more recent studies confirmed the compatibility of adequate soluble heparin doses with chondrogenesis of bone marrow or adipose-derived MSCs [26–28], effects on chondrocyte hypertrophy and specifically type X collagen deposition have not been addressed. Interestingly, soluble heparin reduced growth and differentiation factor (GDF)-5-induced ALP activity in chondrogenic ATDC5 cultures [29], supporting a potential antihypertrophic effect of heparin in vitro.

The aim of this study was to investigate whether heparin–PEG hydrogels or heparin in soluble form can support cell fate instruction during TGF-β-driven MSC in vitro chondrogenesis into the chondral lineage to form stable (nonhypertrophic) neocartilage tissues. To dissect both the biomaterial-mediated and biochemical contributions of heparin to lineage guidance, we chose two complementary approaches. Initially, we immobilized MSCs in the previously established TGF-β1-loaded heparin–PEG hydrogels [18] and cultured these in chondrogenic medium containing additional soluble TGF-β1. To rigorously assess how heparin affects MSC chondrogenesis and hypertrophy, we then utilized the standard chondrogenic pellet culture system (hydrogel-free) in defined chondrogenic medium supplemented with soluble TGF-β1 and increasing concentrations of soluble heparin. We assessed the neocartilage formed after 4 weeks for hallmark characteristics of chondrocyte hypertrophy, including type X collagen deposition, and investigated the influence of soluble heparin treatment on the activation of pro-chondrogenic TGF-β-SMAD2/3 and insulin-AKT pathways, as well as of pro-hypertrophic WNT/β-catenin and SMAD1/5/9 pathways.

## Materials and methods

### Isolation, expansion, and chondrogenic differentiation of human MSCs from bone marrow

Each procedure followed the current revision of the 1975 Helsinki Declaration and was approved by the local Ethics Committee for Human Experimentation of the Medical Faculty of Heidelberg University. After written informed consent, human MSCs were isolated from the bone marrow of patients (28–83 years) undergoing total hip endoprosthesis surgery. A Ficoll-Paque^™^ PLUS (Cytiva, Freiburg, Germany) density gradient was used for mononuclear cell enrichment. Cells were then cultured in 0.1% gelatin-coated culture flasks (37 °C, 6% CO_2_) using an expansion medium composed of high glucose (4.5 g/L) Dulbecco’s modified Eagle medium (DMEM) supplemented with 12.5% fetal bovine serum, 1% penicillin–streptomycin (Pen Strep, Gibco^™^, Thermo Fisher Scientific, Darmstadt, Germany), 2 mM l-glutamine, 1% nonessential amino acids (minimum essential medium), 1% 2-mercaptoethanol (all Gibco^™^, Thermo Fisher), and 4 ng/mL fibroblast growth factor-2 (Active Bioscience, Hamburg, Germany; Miltenyi Biotec, Bergisch Gladbach, Germany). After 24 h, nonadherent cells were removed by washing with phosphate-buffered saline (PBS), and the medium was exchanged thrice weekly.

### Preparation of heparin–PEG hydrogels

The heparin–PEG hydrogels were produced as previously established [21, 30]. To achieve a hydrogel with 50% matrix metalloproteinase (MMP)-sensitive linkers, 0.75 mol non-MMP-sensitive thiol end-functionalized starPEG, 0.75 mol thiol end-functionalized starPEG-MMP conjugates, and 1 mol maleimide-functionalized heparin were reconstituted in PBS and mixed with 1.2 × 10^6^ passage 3 MSCs and TGF-β1 (120 ng per 60 µL hydrogel construct). The hydrogels were then polymerized in 6 mm × 2 mm disc molds and cultured in chondrogenic medium containing high glucose DMEM, 1% Pen Strep, 0.1 μM dexamethasone, 0.17 mM l-ascorbic acid-2-phosphate, 2 mM sodium pyruvate, 0.35 mM proline (all Sigma-Aldrich, Darmstadt, Germany), 1% ITS^™^ + Premix (Corning Life Sciences, New York City, NY, USA), and 10 ng/mL TGF-β1 (PeproTech, Darmstadt, Germany) for 28 days. Since the hydrogels swell in physiological salt solutions, the prepared 22.4 mg/mL heparin in the hydrogel formulation (1.344 mg per 60 µL hydrogel) resulted in ~18 mg/mL heparin in cultured gels. Medium was exchanged thrice weekly.

### Chondrogenic pellet culture

Confluent passage 3 cells were subjected to pellet culture (5 × 10^5^ MSCs) in chondrogenic medium, supplemented as indicated with heparin (10, 100, 700 μg/mL; sodium salt from porcine mucosa; molecular weight (MW): $\approx$ 15,000 Da; Calbiochem, Merck Millipore, Darmstadt, Germany). Medium exchanges were performed three times per week.

### Protein extraction

For collagen extraction, one day-28 pellet per treatment group and donor was digested for 16 h using pepsin (2.5 mg/mL pepsin, 0.5 M acetic acid, 0.2 M NaCl, Carl Roth, Karlsruhe, Germany). After adjusting the pH to 7 using 1 M Tris base (Carl Roth), the collagens were extracted with 4.5 M NaCl (overnight, 4 °C). Of note, this procedure also degrades all typical reference proteins, including β-actin. Collagens were precipitated for 4 h at −20 °C using 100% ethanol. Pelleted collagens were then resuspended in lysis buffer (50 mM Tris, 150 mM NaCl, 1% Triton^™^ X-100, Sigma-Aldrich).

Whole cell lysates were prepared from one to two pellets per treatment group and donor using PhosphoSafe^™^ Extraction Reagent (Merck Millipore) supplemented with 1 mM Pefablock^®^ SC (Sigma-Aldrich) for the detection of SMAD and AKT proteins. For the detection of WNT signaling-associated β-catenin, cells were treated with Saponin buffer (0.05% in Tris-buffered saline and 1 mM MgCl_2_, both Sigma-Aldrich) supplemented with 1 mM Halt^™^ Proteinase/Phosphatase Inhibitor Cocktail (Thermo Fisher Scientific). The extracted supernatant containing both the cytosolic and nuclear pools of β-catenin was mixed with Laemmli buffer (33.2% [w/v] glycerol, 250 mM Tris–HCl pH 6.8 [both Carl Roth], 8.0% [w/v] sodium dodecyl sulfate (SDS), and 0.02% bromophenol blue [both Sigma-Aldrich]). Samples were boiled for 5 min at 95 °C under constant agitation.

### SDS–polyacrylamide gel electrophoresis (PAGE) and western blotting

Collagens were separated using 6% polyacrylamide gels, and all other proteins using 10% gels following standard gel electrophoresis protocols. The proteins were then transferred onto a nitrocellulose membrane (Amersham^™^, GE Healthcare, Chalfont St Giles, UK). Membranes were cut horizontally to detect proteins of different sizes. For collagen detection, the lower part of the membrane was incubated with mouse anti-human type X collagen antibody (1:500, clone X53, no. 41-9771-82, Invitrogen) and the upper part with mouse anti-human type II collagen antibody (1:1000, clone II-4C11, no. 63171, MP Biomedicals, Eschwege, Germany). For the detection of SMAD, AKT, or β-catenin, the upper membrane sections were treated with rat monoclonal anti-pSMAD1/9 (1:1000, pS463/pS465, pS465/pS467, clone N6-1233 (RUO), no. 562509, BD Biosciences, East Rutherford, NJ, USA), rabbit monoclonal anti-pSMAD2 (1:500, pS465/pS467, clone 138D4, no. 3108), rabbit monoclonal anti-pSMAD3 (1:500, pS423/pS425, clone C25A9, no. 9520), rabbit polyclonal anti-pAKT (1:500, pS473, no. 9721, all Cell Signaling Technology, Danvers, MA, USA), or mouse monoclonal anti-β-catenin antibodies (1:10,000, clone 14/Beta-Catenin (RUO), no. 610154, BD Biosciences). The lower membrane sections were probed using mouse monoclonal anti-β-actin (1:10,000, clone AC-15, no. GTX26276, GeneTex, Irvine, CA, USA), or mouse monoclonal anti-CD81 (1:500, clone B-11, no. sc-166029, Santa Cruz Biotechnology, Dallas, TX, USA). For total SMAD detection, membranes were re-incubated with rabbit monoclonal anti-SMAD1/5 (SMAD1: 1:500, clone EP565Y, no. ab33902; SMAD5: 1:1000, EP619Y, no. ab40771; both Abcam, Berlin, Germany) or rabbit monoclonal anti-SMAD2/3 (1:250, clone D7G7, no. 8685). For total AKT detection, rabbit polyclonal anti-AKT (1:100, no. 9272, both Cell Signaling Technology) was used. Secondary antibodies were horseradish peroxidase (HRP)-conjugated with goat anti-rat antibody (1:1000, HAF005, Bio-Techne, R&D Systems, Minneapolis, MN, USA), peroxidase-coupled goat anti-mouse antibody (1:10,000, no. 115-035-071), or peroxidase-coupled goat anti-rabbit antibody (1:10,000, no. 111-035-046, both Jackson ImmunoResearch Laboratories, West Grove, PA, USA). All primary and secondary antibodies were diluted in 5% skim milk in Tris-buffered saline with Tween-20 (TBS-T) (primary: incubated overnight at 4 °C, secondary: 2 h at room temperature). EnhancedChemiLuminescence (ECL) solutions (Roche, Mannheim, Germany) were used for band visualization.

### Type II collagen enzyme-linked immunosorbent assay (ELISA)

The Type II Collagen Detection kit (Chondrex, Woodinville, WA, USA) was used to quantify the type II collagen content in the collagen extraction samples, following the manufacturer’s instructions.

### Glycosaminoglycan and DNA quantification

Per treatment group and donor, one or two day-28 pellets were digested with 0.1 mg/mL proteinase K (in Tris–HCl [50 mM, 1 mM CaCl_2_, Sigma-Aldrich], Thermo Fisher) for 18 h at 60 °C with continuous shaking. Following dilution in Tris/EDTA buffer (Invitrogen, Thermo Fisher Scientific), samples were mixed with 1,9-dimethyl methylene blue solution (48 μM [Thermo Fisher Scientific], in 40 mM NaCl and 40 mM glycine [both Carl Roth]), and the absorbance was measured at 530 nm. The values obtained were referred to a chondroitin 6-sulfate standard curve.

The Quant-iT^™^-PicoGreen^®^ kit (Invitrogen) was used according to the manufacturer’s instructions to quantify the DNA content in the proteinase K-digested samples. The resulting fluorescence values were compared with a λ-DNA-derived standard curve.

### Prostaglandin E2 ELISA

For quantification of the PGE2 content, 48-h conditioned culture supernatants from seven to nine pellets per treatment group and donor were pooled at the indicated time points. Following the provider’s protocol, a colorimetric competitive PGE2 ELISA (Enzo Life Sciences, Farmingdale, NY, USA) was performed.

### ALP enzyme activity assay

The supernatants from seven to nine pellets per treatment group and donor were pooled at the indicated time points. Alternatively, one pellet or half a heparin–PEG hydrogel sample was lysed using Triton X-100 (1% in PBS, Sigma-Aldrich). An equal volume of *p*-nitrophenyl phosphate substrate (10 mg/mL in 0.1 M glycine [Carl Roth], 1 mM MgCl_2_, and 1 mM ZnCl_2_ [Sigma-Aldrich], pH 9.6 for supernatant testing or pH 10.4 for cell lysates) was added. After 120 min, the substrate conversion was detected at 405 nm and corrected by the signal at 490 nm. The enzyme activity was calculated using a standard curve derived from *p*-nitrophenol (Sigma-Aldrich).

To normalize the ALP activity obtained from cell lysates, the Pierce^™^ BCA Protein Assay kit (Thermo Fisher) was used according to the manufacturer’s instructions to quantify the protein content in the cell lysates. The absorbance was measured at 562 nm, and the resulting values were compared with a bovine serum albumin-derived standard curve.

### Analysis of gene expression

Three day-28 pellets were pooled and homogenized for each group and donor using a polytron. RNA was extracted from the homogenate using TRIzol^®^ reagent (Invitrogen) following the manufacturer’s instructions. Complementary DNA was synthesized from 500 ng total RNA using oligo-dT primers and the OmniScript^®^ Reverse Transcription kit (QIAGEN, Hilden, Germany). SYBR^®^ Green master mix (Thermo Fisher), specific primer sequences (Supplementary Table S1), and a LightCycler^®^ 96 device (Roche Diagnostics, Basel, Switzerland) were used to determine the transcript levels of the genes of interest via quantitative PCR (qPCR). Relative gene expression was calculated as 1.8^−ΔCt^ as described before [31], with ΔCt representing the difference between the Ct value of the gene of interest and the mean Ct values of the reference genes *CPSF6* and *HPRT*. The quality of the qPCR products was evaluated by melting curve analysis and agarose gel electrophoresis.

### Immunohistochemistry

Day-28 samples were fixed in 4% formaldehyde (in PBS, pH 7.4), dehydrated through a graded propane-2-ol series, and embedded in paraffin. From these, 5-μm-thin microsections were generated. Following deparaffinization and rehydration, sections were treated with hyaluronidase (4 mg/mL in PBS, pH 5.5). Thereafter, sections were incubated with either pronase (1 mg/mL in PBS, pH 7.4, both from Roche Diagnostics, Mannheim, Germany) for type II collagen immunostaining, or with proteinase XXIV (0.02 mg/mL in PBS, pH 7.4, Sigma-Aldrich) for type X collagen or aggrecan immunostaining. For type X collagen immunostaining in hydrogels, pepsin (4 mg/mL in 0.01 M NaOH, Sigma-Aldrich) was used instead of proteinase XXIV. Subsequently, sections were blocked with 5% bovine serum albumin. Human type II collagens, type X collagens, or aggrecans were visualized after incubation with a mouse anti-type II collagen antibody (1:1000, same as for western blotting), a mouse anti-type X collagen antibody (1:1, as above), and a mouse anti-aggrecan antibody (1:25, clone HAG7D4 (7D4), no. SM1353, Acris/OriGene, Rockville, MD, USA), respectively, and an ALP-coupled goat anti-mouse immunoglobulin G secondary antibody (ImmunoLogic, WellMed, MS Arnhem, the Netherlands) using the ImmPACT^®^ Vector^®^ Red Substrate kit (Vector Laboratories, Newark, CA, USA). Alkaline phosphatase activity in deparaffinized and rehydrated microsections was detected using NBT/BCIP substrate solution (in Tris–HCl [1 M, pH 9.5]; Merck).

As positive, negative, and isotype staining controls (Supplementary Fig. S2), microsections of previously characterized day-28 MSC chondrogenic cultures (pellets or heparin–PEG hydrogels) from earlier independent studies were chosen. For isotype controls, primary antibodies were replaced with 1% bovine serum albumin in PBS; otherwise, all control microsections were treated identically to experimental samples.

### Statistical methods

The figure captions indicate the number of independent biological replicates for each experiment. Data were analyzed using SPSS Statistics (version 29.0.0.0, IBM, Armonk, NY, USA). The Mann–Whitney *U* test was used to evaluate differences between groups. Box plots represent the interquartile range (IQR) extending between the 25th and the 75th percentiles (Q1, Q3), and lines inside the boxes represent the median. Whiskers extend to minimum and maximum values. Black circles in the graphs represent the single data points. Extreme outliers, indicated as empty circles, were determined using Tukey’s fences: Q3 $+$ 3(Q3 $-$ Q1). A paired Student’s *t*-test was used for time-course studies. Results were considered statistically significant at *p* $\le$ 0.05.

## Results

### Endochondral commitment of MSCs in heparin–PEG hydrogels in vitro

**Fig. 1:**
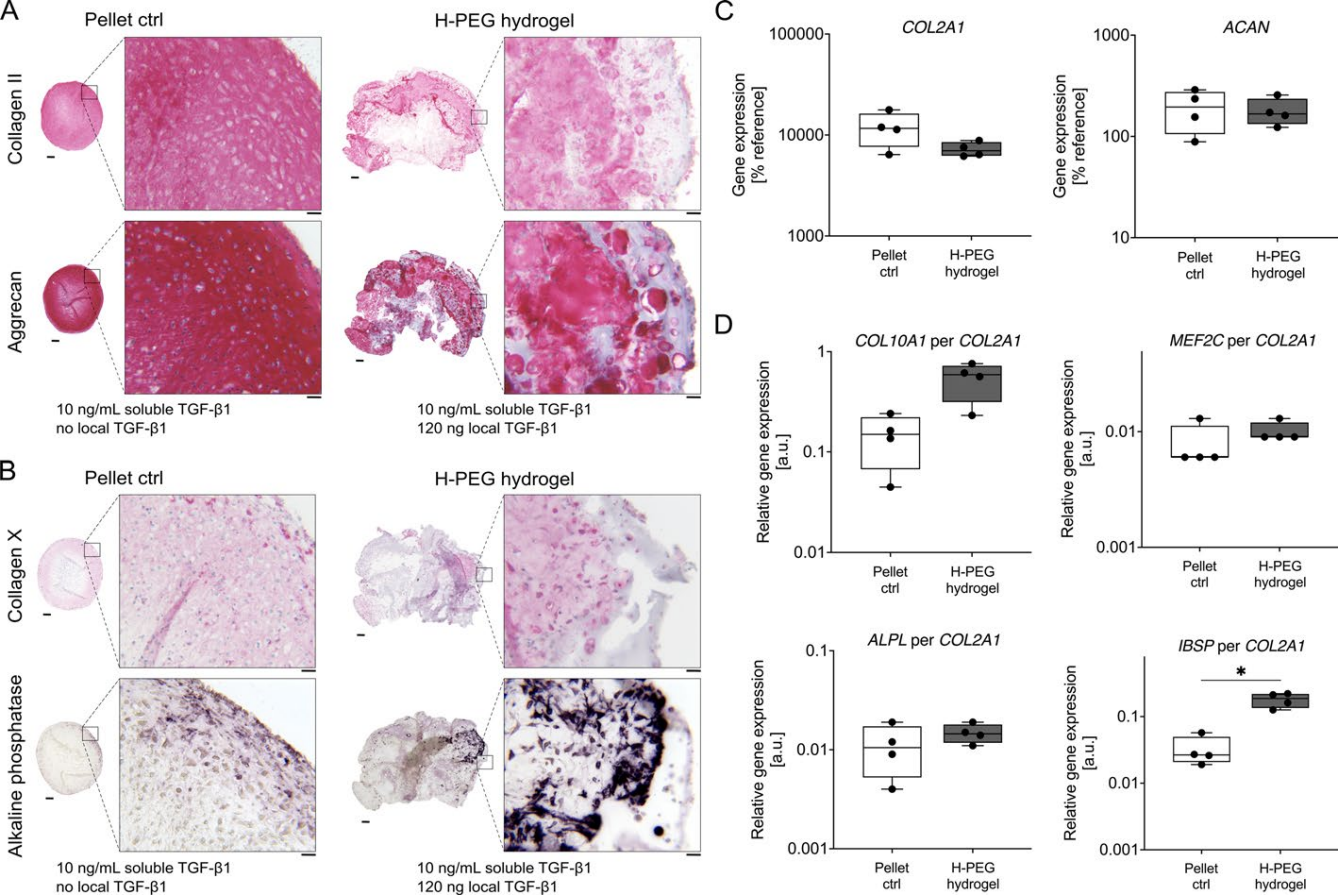
MSC in vitro chondrogenesis in heparin–PEG hydrogels versus pellet controls. MSCs were cultured as pellets (pellet control [ctrl]; 5 × 10^5^ cells) or in heparin–PEG hydrogels (H–PEG hydrogel; 1.2 × 10^6^ cells) containing 22.4 mg/mL crosslinked heparin and 120 ng TGF-β1 for 4 weeks in TGF-β1-containing (10 ng/mL) standard chondrogenic medium in vitro. **A**, **B** Microsections of day-28 samples were assessed either via immunohistochemistry to detect type II collagen or aggrecan, and type X collagen, or by enzymatic activity staining to visualize alkaline phosphatase, as indicated (scale bar: overview = 200 µm, magnification = 50 µm; positive and negative staining controls are shown in Supplementary Fig. S2). One representative result out of *n* = 5 experiments with independent MSC donor populations is shown. **C** Day-28 gene expression levels of chondrocyte and **D** hypertrophy markers, with *CPSF6* and *HPRT* used as reference genes. The hypertrophy markers in day-28 samples are expressed as ratio to *COL2A1*. *n* = 4 experiments using independent MSC donor populations. Box plots were built as described in the statistics section. ^*^p $\le$ 0.05, Mann–Whitney *U* test

An initial pilot experiment indicated that MSC chondrogenesis in heparin–PEG hydrogels loaded with TGF-β was improved by additional exogenous TGF-β (10 ng/mL) supplementation of the chondrogenic medium (Supplementary Fig. S1). Next, we investigated whether soluble TGF-β addition induced a reproducibly strong deposition of cartilaginous matrix by MSCs. We prepared hydrogels with the same composition as in our previous study (60 µL PEG hydrogel formulation containing 22.4 mg/mL crosslinked heparin, 120 ng TGF-β1, 1.2 × 10^6^ MSCs) [18] and cultured them in standard TGF-β1-containing chondrogenic medium (10 ng/mL). Standard pellet cultures (hydrogel-free) comprising 0.5 × 10^6^ MSCs without local TGF-β were used as controls. After 4 weeks, we detected substantial deposition of type II collagen and the main cartilage proteoglycan aggrecan in the hydrogels by histological analysis in all five independent experiments (Fig. 1A). The cartilage matrix remained pericellular only in few areas, and not all cells had differentiated into chondrocytes, consistent with our previous in vivo results [18]. In control pellets and in the heparin group, strong and homogeneous deposition of type II collagen and aggrecan was detected, and sufficient neocartilage was obtained to examine chondrocyte hypertrophy.

We next addressed whether MSCs committed to chondral or endochondral differentiation in heparin–PEG hydrogels in vitro. Both the control pellets and the obtained neocartilage in the heparin–PEG group stained positive for type X collagen as well as the mineralizing enzyme ALP (Fig. 1B). Importantly, these data indicate that chondrocytes became hypertrophic regardless of the presence of PEG-immobilized heparin when cultured in standard chondrogenic medium. Of note, the MSC-derived neocartilage tissue that had formed in subcutaneous pouches in heparin–PEG hydrogels in our previous study was fully devoid of type X collagen and ALP [18], indicating that the lineage-instructive ability of heparin-coupled TGF-β is context-dependent.

To further support our histological observations, we assessed gene expression levels of chondrocyte and hypertrophy markers (Fig. 1C,D). Expression for both *COL2A1* and *ACAN* was detectable in heparin–PEG hydrogels and control samples. For hypertrophy markers, expression data were referred to *COL2A1* expression to adjust for different chondrocyte quantities between pellet controls and hydrogels. In line with histological observations, qPCR analysis revealed strong expression of *COL10A1* and *ALPL* mRNAs along with *MEF2C* and *IBSP* in the heparin–PEG hydrogel group that was at least as high as in pellet controls (Fig. 1C,D and Supplementary Fig. S3A–C). In agreement with our histological data, ALP enzyme activity was detectable in cell lysates from both pellets and heparin–PEG hydrogels (Supplementary Fig. S3D). Thus, overall, MSCs underwent hypertrophic misdifferentiation in heparin–PEG hydrogels in standard chondrogenic medium, and heparin failed to redirect TGF-β-driven MSC chondrogenesis toward the desired chondral lineage in vitro.

Of note, additional exogenous supplementation of 10 ng/mL TGF-β1 was not responsible for the hypertrophic differentiation of MSCs in heparin–PEG hydrogels, since type X collagen was also detected in MSC-laden heparin–PEG hydrogels cultured in the absence of soluble TGF-β1 (Supplementary Fig. S4A). Additionally, reducing the TGF-β1 dose in the chondrogenic medium of MSC pellets resulted in reduced proteoglycan accumulation but failed to prevent type X collagen deposition or ALP expression (Supplementary Fig. S4B,C).

### Effects of soluble heparin on lineage-directive cell signaling pathways

Modulating the heparin concentration in the hydrogel will inevitably change the mobility of TGF-β in the gel, its availability to the cells, and therefore chondrogenic differentiation. Thus, the effect of heparin on cell signaling and chondrocyte hypertrophy could not be addressed rigorously in this approach. Therefore, we next employed the common pellet culture system, which allowed us to assess the dose-dependent effects of soluble heparin on chondrogenesis and hypertrophy. First, we investigated the influence of soluble heparin on the activation of pro-chondrogenic and pro-hypertrophic signaling pathways at the initiation of chondrogenesis. Serum-free, defined standard chondrogenic medium with or without 10 ng/mL soluble TGF-β1 and 6.25 μg/mL insulin was preincubated for 60 min with free soluble heparin at concentrations of 0, 10, 100, or 700 μg/mL and then used to stimulate pelleted passage 3 MSCs. As established [18], 700 μg/mL heparin was used as the maximum concentration. The 22.4 mg/mL heparin-containing PEG hydrogel formulation could not be tested in this experimental set-up owing to its cytotoxic effects.

**Fig. 2:**
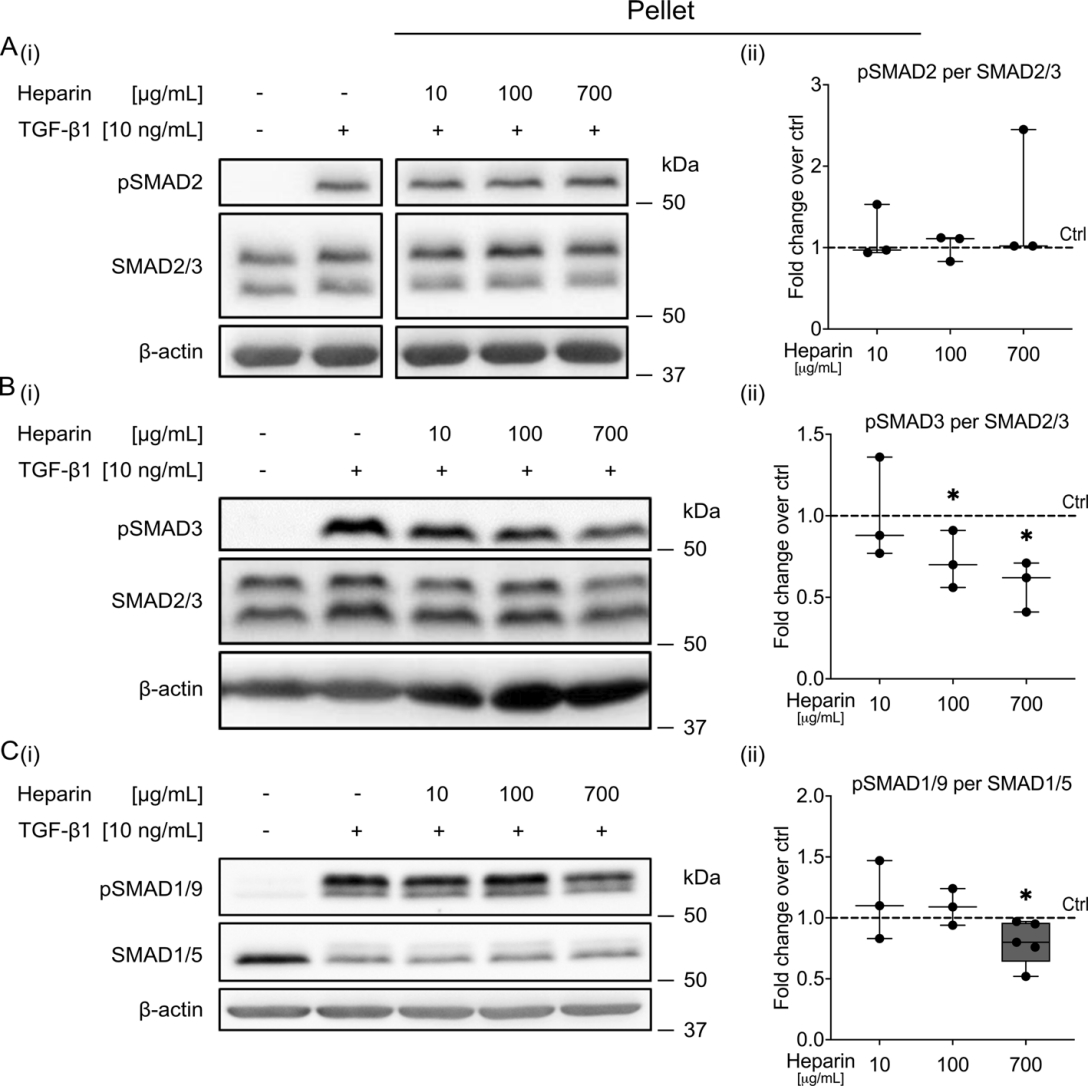
Effect of soluble heparin on TGF-β-induced SMAD activation in MSC pellets in vitro. Serum-free, defined chondrogenic medium, with or without TGF-β1 (10 ng/mL), was preincubated with soluble heparin (0, 10, 100, 700 µg/mL) for 60 min and then added to 5 × 10^5^ pelleted passage 3 MSCs. After 30 min, cells were harvested and whole protein lysates were prepared for western blotting. **A** Phospho-SMAD2 and total SMAD2/3, **B** phospho-SMAD3 and total SMAD2/3, or **C** phospho-SMAD1/9 and total SMAD1/5 were detected. β-actin was used as an additional internal reference (all blots are shown in Supplementary Fig. S5; uncropped blot pictures are provided as Supplementary Material S1). All samples in each line were run on the same gel and blotted onto one membrane. One out of *n* = 3–5 experiments using independent MSC donor populations is shown. Densitometric analysis of western blots was normalized to total SMAD2/3 or total SMAD1/5, and control samples (+TGF-β1, no heparin) were each set to 1 (^*^p $\le$ 0.05 versus control, Mann–Whitney *U* test). Box plots were built as described in the statistics section

Western blotting showed that pro-chondrogenic SMAD2 phosphorylation was induced by TGF-β1 irrespectively of the presence of any tested heparin concentration (Fig. 2A and Supplementary Fig. S5A). By contrast, heparin dose-dependently decreased TGF-β1-induced pro-chondrogenic SMAD3 activation in all three examined donor populations (Fig. 2B and Supplementary Fig. S5B). A slight SMAD2/3 dichotomy was also observed in samples harvested after 28 days of chondrogenesis (Supplementary Fig. S6A,B). Reduced SMAD3 activation under heparin treatment was in line with the previously observed reduction of TGF-β reporter activity in MSCs within heparin–PEG hydrogels compared with heparin-free controls [18]. Importantly, SMAD1/9 phosphorylation, which was induced by TGF-β1 at initiation of chondrogenesis, was largely unaffected by 10 and 100 μg/mL heparin. It was, however, significantly decreased by 700 μg/mL heparin (Fig. 2C and Supplementary Fig. S5C). On day 28, no obvious reduction of TGF-β1-SMAD1/5/9 activation by any tested heparin concentration was observed (Supplementary Fig. S6C). Remarkably, this is in contrast to suppressed pSMAD1/5/9 levels, which were previously observed in vivo [18].

**Fig. 3:**
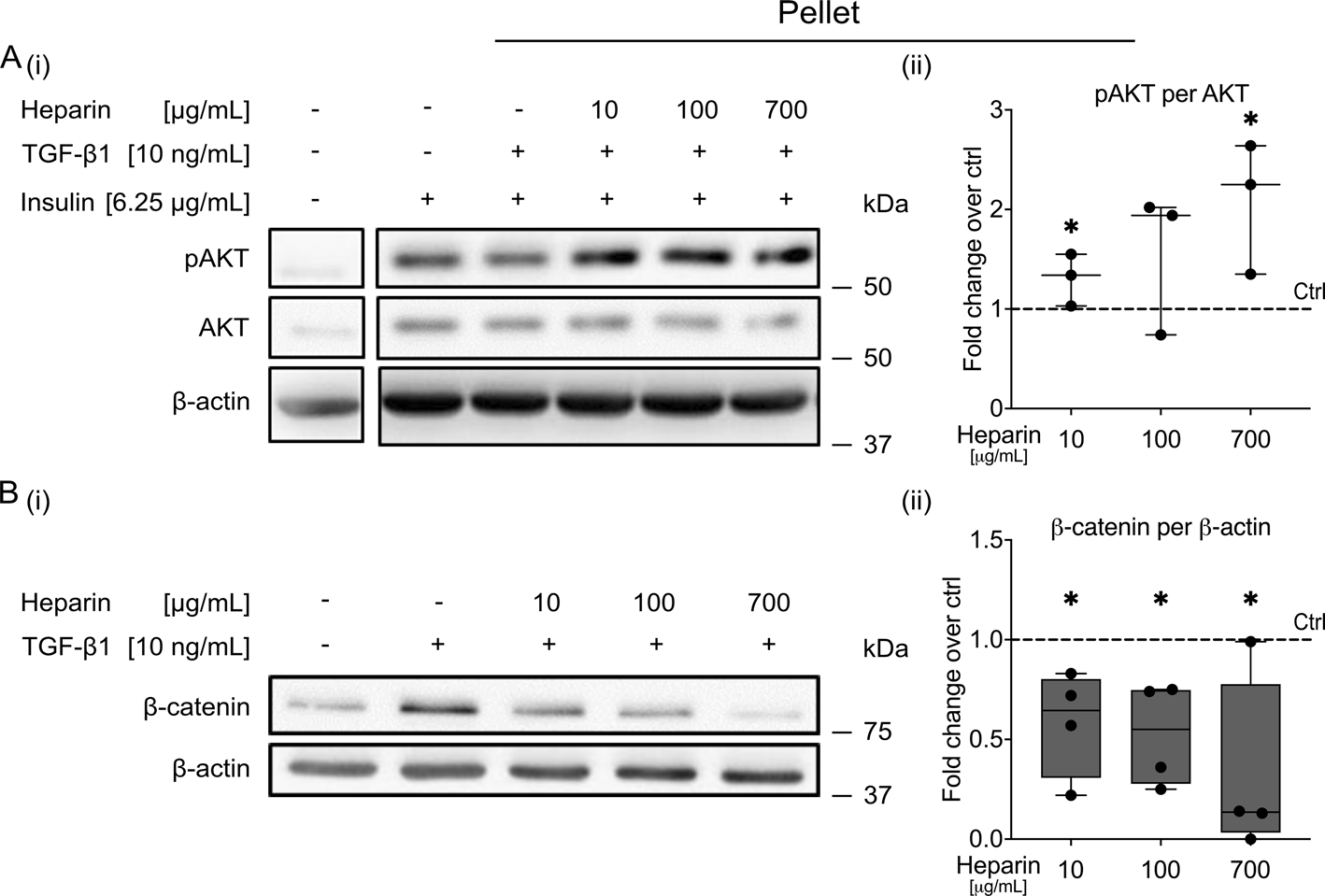
Effect of soluble heparin on AKT activation and β-catenin accumulation in MSC pellets in vitro. Serum-free, defined chondrogenic medium, with or without TGF-β1 (10 ng/mL) and with or without insulin (6.25 µg/mL) was preincubated with soluble heparin (0, 10, 100, 700 µg/mL) for 60 min and then added to 5 × 10^5^ pelleted passage 3 MSCs. **A** After 30 min, samples were harvested and whole protein lysates were analyzed for phospho-AKT and total AKT using western blotting. All samples in each line were run on the same gel and blotted onto one membrane.** B** Membrane-depleted extracts of 3-h samples were interrogated for β-catenin protein levels. β-actin was used as an internal reference. Representative blots of *n* = 3–4 independent experiments with independent MSCs are shown (all blots are shown in Supplementary Fig. S7; uncropped blot pictures are provided as Supplementary Material S1). Box plots were built as described in the statistics section with a dashed line representing control samples set to 1 (+TGF-β1, +insulin, no heparin). ^*^p $\le$ 0.05 versus control, Mann–Whitney *U* test

Additionally, heparin supplementation mildly increased the insulin-induced phosphorylation of pro-chondrogenic AKT significantly (Fig. 3A and Supplementary Fig. S7A), which was previously shown to be important for TGF-β1-induced SMAD2 phosphorylation [4]. In turn, TGF-β1-stimulated pro-hypertrophic β-catenin accumulation was significantly reduced by all tested heparin concentrations (Fig. 3B and Supplementary Fig. S7B), which was in line with our previous in vivo observations.

Taken together, soluble heparin exhibited a surprising dichotomous effect on TGF-β/SMAD2/3 activation, inhibiting SMAD3 but not SMAD2 phosphorylation, which was possibly facilitated by enhanced insulin/AKT signaling. Concurrently, soluble heparin treatment reduced the activation of the endochondral WNT/β-catenin pathway but lowered SMAD1/5/9 activation only mildly and transiently. Together, these data suggested that soluble heparin supplementation during MSC in vitro chondrogenesis may reduce differentiation into chondrocytes and exhibit a mild antihypertrophic activity.

### Effect of soluble heparin on MSC in vitro chondrogenesis

We then investigated whether the observed maintained SMAD2 but reduced SMAD3 activation would reduce MSC in vitro differentiation into chondrocytes. Therefore, we determined gene expression levels of chondrocyte markers after 28 days of chondrogenic culture. The expression of *COL2A1*, *ACAN*, and *SOX9* was strongly and significantly increased in the untreated control and 10 µg/mL heparin groups compared with day-0 samples (Supplementary Fig. S8A–C). Only referencing to individual controls to account for donor variability of MSC populations revealed small but significant reductions of *SOX9* and *ACAN* expression, but not *COL2A1*, whereas *COMP* was significantly enhanced by 10 µg/mL heparin (Fig. 5A). Following treatment with 100 µg/mL or 700 µg/mL heparin, we observed a dose-dependent increase in median Ct values for all tested reference genes, and for the robust *CPSF6* and *HPRT*, this became significant at 700 µg/mL (Supplementary Fig. S8D), indicating that soluble heparin treatment interfered with RNA isolation. Thus, the implications of gene expression analyses in the 100 and 700 µg/mL heparin groups remain unclear, and the data are therefore not included in the manuscript.

**Fig. 4:**
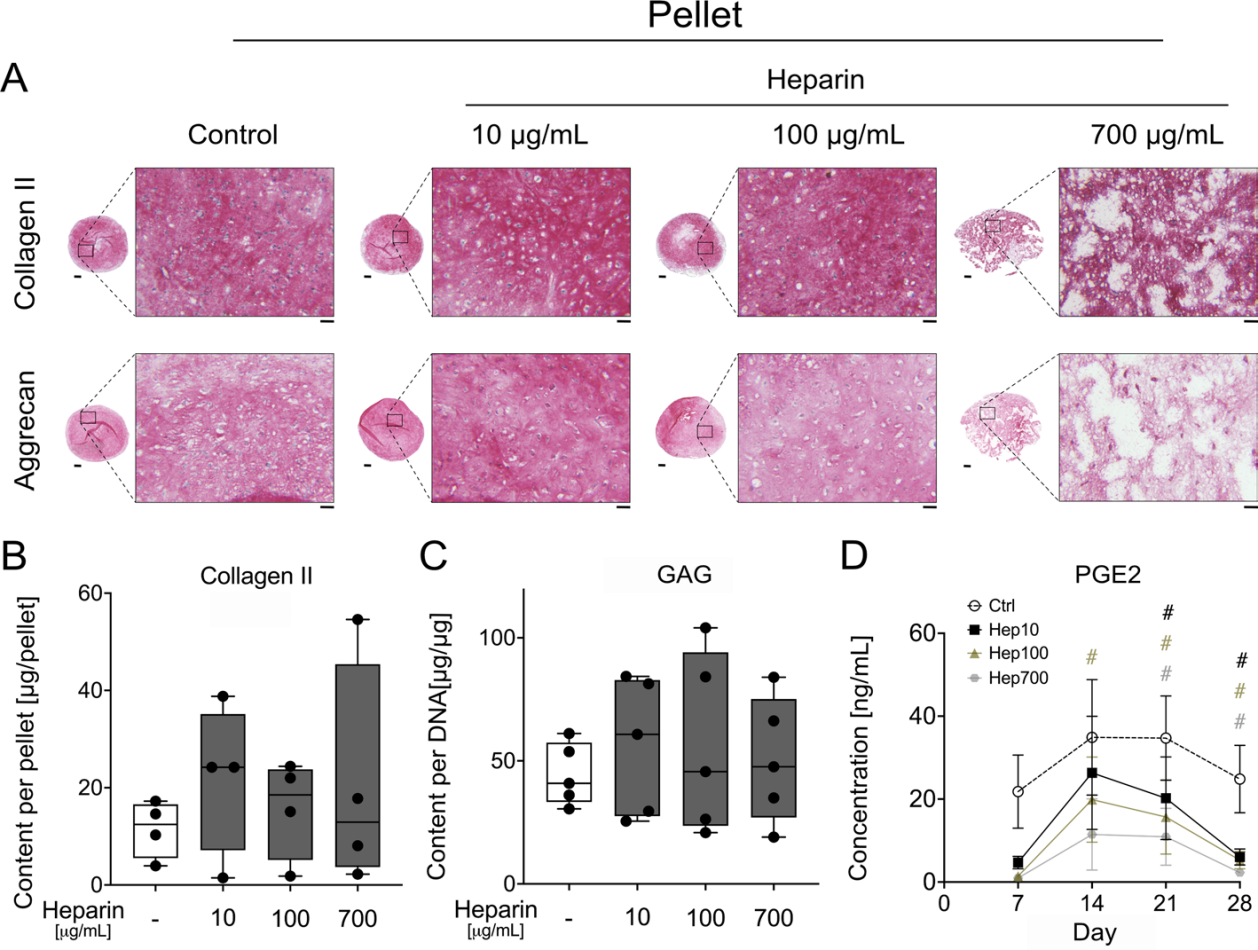
Effect of soluble heparin on MSC in vitro chondrogenesis. MSCs were cultured as pellets for 28 days in standard chondrogenic medium (including 10 ng/mL TGF-β1 and 6.25 µg/mL insulin) supplemented with 0, 10, 100, or 700 µg/mL soluble heparin. **A** Paraffin microsections of day-28 samples were assessed for type II collagen and aggrecan via immunohistochemistry (scale bar: overview = 200 µm, magnification = 50 µm). Representative pictures of *n* = 5 experiments with independent MSCs are shown. **B** Type II collagen content per pellet was measured by ELISA (*n* = 4). **C** Proteoglycan content per pellet was assessed using DMMB assay and normalized to DNA amounts per pellet (*n* = 5). **D** Cell culture supernatants were collected from seven to nine pellets at weekly intervals and analyzed for PGE2 levels using ELISA. Box plots were built as described in the statistics section and analyzed using Mann–Whitney *U* test. No statistically significant differences were found between the groups (*p* $>$ 0.05). Line graphs show data as mean $\pm$ standard error of the mean (SEM). ^#^*p* $\le$ 0.05 versus control, paired Student’s *t*-test

We next evaluated the neocartilage matrix accumulated during 4 weeks of chondrogenesis to investigate possible effects of soluble heparin on cartilage formation. Immunohistochemical and histological analyses revealed a strong and homogeneous deposition of type II collagen and aggrecan (Fig. 4A). Cells showed the typical round chondrocyte morphology in all groups. However, the integrity of the extracellular matrix appeared compromised by 700 μg/mL heparin in all tested MSC donor samples and by 100 μg/mL heparin in two out of five MSC donors. ELISA, dimethylmethylene blue (DMMB), and PicoGreen assays showed similar quantities of type II collagen, GAG/DNA, and DNA per pellet, respectively (Fig. 4B,C and Supplementary Fig. S8E), demonstrating similar levels of chondrogenic differentiation and matrix deposition among all groups.

Prostaglandin E2 (PGE2) was previously identified as an autocrine antihypertrophic mediator upregulated during MSC in vitro chondrogenesis [31]. ELISA revealed that PGE2 levels in cell culture supernatants were dose-dependently decreased by heparin throughout MSC chondrogenesis (Fig. 4D), reaching −87 ± 2% compared with the control group at culture termination (Supplementary Fig. S8F). Adding heparin to PGE2 ELISA standards confirmed that heparin did not disturb PGE2 detection (Supplementary Fig. S8G). Overall, while allowing strong chondrogenesis and cartilage formation at a low dose, increasing concentrations of soluble heparin compromised neocartilage integrity and strongly reduced levels of antihypertrophic PGE2. The high content of cartilaginous matrix in the pellets of all groups demonstrated maintained chondrogenic differentiation despite reduced pSMAD3 levels, suggesting that the increased activation of AKT and sustained SMAD2 induction could compensate for the reduced SMAD3 induction.

### Soluble heparin is selectively antihypertrophic in vitro

**Fig. 5:**
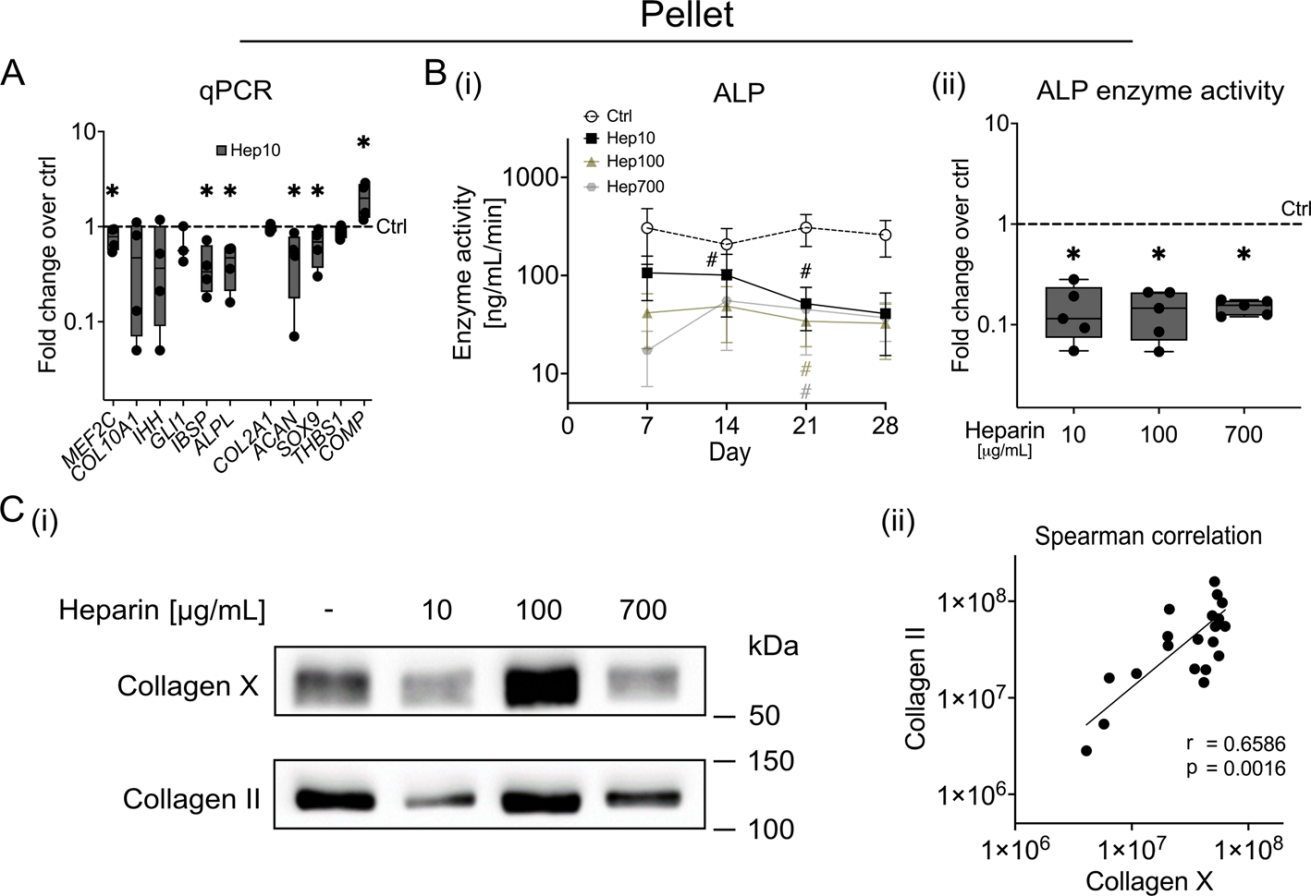
Effect of soluble heparin on hypertrophy during MSC chondrogenesis in vitro. MSC pellets were cultured for 4 weeks in standard chondrogenic medium (including 10 ng/mL TGF-β1 and 6.25 µg/mL insulin) containing soluble heparin (0, 10, 100, or 700 µg/mL as indicated). **A** Gene expression levels of hypertrophy markers and TGF-β-responsive genes as indicated, with *CPSF6* and *HPRT* used as reference genes (*n* = 3–4) and referred to untreated controls. Only data from the 10 µg/mL heparin group are shown. Data from the 100 and 700 µg/mL groups were excluded owing to compromised RNA isolation. **B** ALP enzyme activity was determined in the pooled supernatants of seven to nine MSC pellets at weekly intervals (*n* = 5). Data are shown as mean $\pm$ SEM. ^#^*p* $\le$ 0.05 versus control, paired Student’s *t*-test. **C(i)** Type X collagen content was determined via western blotting in pepsin-digested day-28 MSC pellets, with type II collagen used as a reference. Representative blots of *n* = 5 from four independent donors are shown; all blots are depicted in Supplementary Fig. S10. **C(ii)** Histogram of type X collagen versus type II collagen band intensities from western blotting was analyzed by Spearman’s correlation, with the *r*-value and *p*-value indicated. Box plots were built as described in the statistics section. ^*^p $\le$ 0.05 versus control, Mann–Whitney *U* test

Next, we assessed chondrocyte hypertrophy after 4 weeks of differentiation. As expected, the expression of the pro-hypertrophic transcription factor *MEF2C* along with *COL10A1*, *IHH*, the Hedgehog target gene *GLI1*, and *IBSP* mRNAs were significantly upregulated in control pellets compared with day 0 (Supplementary Fig. S9A). Of note, treatment with 10 µg/mL heparin slightly but significantly reduced the day-28 levels of *MEF2C*, *IBSP,* and *ALPL* mRNAs relative to control pellets (dashed line), while the reduction of *COL10A1*, *IHH*, and *GLI1* expression in all but one donor failed to reach significance (Fig. 5A). Whether heparin reduced the expression of these markers in a dose-dependent manner remained inconclusive, as the technical limitations described above prevented reliable interpretation of the gene expression data obtained from the 100 µg/mL and 700 µg/mL heparin groups. Of note, typical TGF-β-responsive genes, including *COL2A1*, *SOX9*, *ACAN*, *THBS1*, and *COMP* showed no consistent regulation (Fig. 5A). In addition, the expression of BMP/TGF-β receptors *ALK2* and *ALK4* was detectable at both day 0 and day 28 of differentiation, and no significant changes were observed in response to heparin treatment (Supplementary Fig. S9B). Importantly, quantitative assessment of ALP enzyme activity in cell culture supernatants showed that all heparin concentrations strongly decreased secreted ALP levels during MSC chondrogenesis (Fig. 5B(i)). On day 28, the mean reduction reached 85% dose-independently in all groups compared with controls (Fig. 5B(ii)). To rule out that the presence of heparin interfered with ALP detection, we added 10–700 µg/mL heparin to supernatants of control pellets. However, we found no effect on the detected enzyme activity (Supplementary Fig. S9C). Importantly, western blotting of pepsin-digested samples showed that type X collagen protein levels were not reproducibly reduced by heparin but rather strictly correlated with type II collagen protein levels (Fig. 5C and Supplementary Fig. S10). Of note, pepsin digestion is necessary to quantitatively release collagens from the strongly crosslinked cartilaginous matrix. It also degrades all proteins that are commonly used as reference, including β-actin and GAPDH.

Collectively, our findings indicated that under standard in vitro chondrogenesis conditions, neither PEG-immobilized heparin nor heparin in soluble form was capable of redirecting MSC fate into the chondral instead of the endochondral cell lineage. Yet, heparin was selectively antihypertrophic and strongly reduced the mineralizing enzyme ALP, albeit the important hypertrophy marker type X collagen remained unresponsive. Of note, this coincided with a WNT/β-catenin inhibition but ineffective silencing of TGF-β-mediated SMAD1/5/9 activation by heparin, as well as a reduction of antihypertrophic PGE2 levels throughout differentiation.

## Discussion

Preventing endochondral ossification and maintaining a stable chondral phenotype are key to achieving lasting, functional cartilage repair with MSC-based therapies. The currently most effective stabilizing strategies in vitro, namely PTHrP pulse treatment and WNT inhibition, are only selectively antihypertrophic and fail to inhibit type X collagen production [8, 17]. Conversely, in a subcutaneous mouse model, heparin–PEG hydrogels loaded with TGF-β silenced WNT and SMAD1/5/9 pathways and permitted formation of permanent neocartilage from MSCs that was fully devoid of type X collagen and exhibited long-term resistance to calcification and bone formation [18]. The current in vitro study now demonstrates that critical, but so far unknown, contributions originating from the in vivo environment were mandatory to allow heparin–PEG to guide MSCs into the chondral instead of the endochondral lineage.

A thorough comprehension of the molecular mechanisms underlying the chondral versus endochondral lineage decision of MSCs is a prerequisite for controlling their directed differentiation both in fully defined conditions and after orthotopic implantation in the joint. For articular cartilage regeneration, in vitro chondrogenic predifferentiation of MSCs is advantageous as it allows for quality control and has shown significantly better histologic scores and morphologic characteristics of hyaline cartilage in large animal studies [1, 2]. Selecting sufficiently predifferentiated grafts can mitigate the known variability of MSCs [32], thus ensuring reproducible therapy outcomes. Furthermore, the preformed cartilaginous extracellular matrix can increase the graft robustness and protect the cells from overload or toxic inflammation after implantation, thereby reducing failure rates in more challenging surgical settings. Directing MSCs toward a chondral instead of an endochondral cell fate during in vitro predifferentiation is, therefore, a key requirement for the effective use of MSCs in future cartilage repair therapies.

For our initial investigations, we chose the same heparin–PEG hydrogel formulation used in our prior in vivo experiments to address the ability of the biomaterial to instruct MSCs into the chondral lineage under defined standard TGF-β-driven in vitro chondrogenesis conditions. Since modulating heparin concentration in the hydrogel affects TGF-β mobility, availability, and chondrogenesis speed, its impact on cell signaling and chondrocyte hypertrophy was not assessed in this setup. We therefore leveraged the conventional pellet culture system to assess the dose-dependent effects of soluble heparin on chondrogenesis and hypertrophy. This system allowed for a clear, controlled comparison between heparin-free conditions and varying concentrations of soluble heparin, which was essential for establishing a dose–response relationship and evaluating the intrinsic antihypertrophic potential of heparin in a standardized context. To use two complementary approaches was a deliberate and synergistic strategy to dissect both the biochemical (soluble heparin effects) and biomaterial-mediated contributions (immobilized heparin effects) to lineage guidance and was important to demonstrate that the immobilized high-dose heparin within the PEG hydrogels cannot overcome limitations of the antihypertrophic potential of soluble heparin in vitro.

We showed here for the first time that heparin reduced the activation of the pro-hypertrophic WNT/β-catenin signaling pathway and exhibited antihypertrophic activity during in vitro MSC chondrogenesis. By lowering the levels of the mineralizing enzyme ALP strongly (by 85%), already in its lowest applied concentration (10 µg/mL), heparin reached the effectiveness of PTHrP pulse treatment and WNT inhibition during chondrogenesis [8, 9, 17]. In addition, its potency to reduce mean *IBSP* (−60%) and *IHH* expression (−51%) was within the range of previous results with PTHrP pulses and WNT inhibition [8]. Higher heparin concentrations of 100 µg/mL and beyond were, however, not advantageous because they compromised pellet integrity. Thus, we suggest to use heparin in a 10 µg/mL dose as antihypertrophic intervention in future experiments. Notably, WNT inhibition and daily pulses with PTHrP require either the application of expensive proteins or may induce adverse effects due to solvents (e.g., DMSO); or they require complex and cumbersome application protocols. By contrast, heparin is cost-effective and can simply be applied with the routine medium (three times a week). Moreover, its extensive clinical application ensures access to high-quality grades and formulations; regulatory hurdles are well-mapped, and downstream translational applications are greatly facilitated.

One of the most important findings of this study was the observation of a dichotomous regulation of TGF-β-mediated SMAD2/3 activation in response to heparin treatment. While we observed SMAD3 regulation only at heparin concentrations higher than 10 µg/mL, it is likely that this phenomenon occurs over a broader concentration range, but was not detectable owing to the limitations in sensitivity of western blotting. The dichotomous response was surprising, since SMAD2 and SMAD3 are mostly reported to be co-regulated. Consistent with this observation, our previous assays with the SB4 reporter showed a decreased SMAD3 reporter activity by TGF-β in MSCs cultured in the heparin–PEG hydrogels compared with heparin-free controls [18]. Obviously, heparin modulates the interaction of TGF-β with its receptors in a way that less SMAD3 is activated in vitro, while SMAD1/5/9 activity is especially silenced in vivo. Since we previously demonstrated that insulin-AKT signaling enhances TGF-β-mediated SMAD2 phosphorylation during MSC chondrogenesis in vitro [4], we propose here that the stimulation of insulin-AKT activation by heparin supported SMAD2 activation by TGF-β. Importantly, reduced overall SMAD3 activation did not impair MSC differentiation into chondrocytes according to unaffected amounts of extracellular matrix components in the neocartilage tissue. Thus, we propose that reduced SMAD3 activity can be tolerated when SMAD2 is sufficiently activated. This interpretation is consistent with the previous observation that genetic ablation of *Smad2* affected mouse growth plate chondrocytes more severely than loss of SMAD3, suggesting that SMAD2 plays a more prominent role than SMAD3 in chondrogenesis [33].

The lack of robust MSC in vitro chondrogenesis in heparin–PEG hydrogels containing immobilized TGF-β1 in the absence of soluble TGF-β1 strongly suggested that the growth factor was sequestered, and its bioavailability for receptor binding was reduced. Indeed, the high affinity of heparin for TGF-β1 has been extensively documented, with well-characterized binding domains on the TGF-β1 protein [19, 34]. Soluble heparin modestly reduced TGF-β1-induced phosphorylation of SMAD3 and SMAD1/5/9, as well as β-catenin accumulation, and these findings were consistent with sequestration of TGF-β1 even in solution. Notably, we previously reported that TGF-β1 inhibits insulin-induced AKT activation during MSC chondrogenesis [4]. Therefore, the observed increase in AKT phosphorylation in the presence of heparin was consistent with the relief of this inhibition—i.e., owing to reduced TGF-β1 bioavailability. All findings presented here are thus consistent with heparin-mediated sequestration of TGF-β1. Although heparin has been reported to protect TGF-β1 from degradation in some contexts (e.g., via inhibition of α_2_-macroglobulin-mediated inactivation; [34, 35]), and MSCs produce α_2_-macroglobulin ([36, 37] and our own unpublished transcriptomic data), our data indicate that the net outcome was a loss of TGF-β bioactivity. This suggested that sequestration dominated over potential stabilization in our system. Taken together, we propose that heparin-mediated sequestration of TGF-β1 is the key mechanism underlying the observed dichotomous regulation of SMAD2/3 and antihypertrophic activity of heparin during in vitro chondrogenesis of mesenchymal stromal cells.

Yet, similar to all other current antihypertrophic in vitro interventions, treatment with heparin remained only selectively effective and did not prevent MSCs from committing to endochondral development. Yet again, upregulation of the calcium-binding type X collagen that identifies the hypertrophic chondrocyte zone in the growth plate proved unresponsive to antihypertrophic treatment. By contrast, neocartilage that was fully devoid of type X collagen formed in heparin–PEG hydrogels loaded with TGF-β in vivo [18]. We previously proposed the balance between SMAD2/3 and SMAD1/5/9-mediated TGF-β signaling as one possible director of chondral versus endochondral lineage instruction of MSCs [18, 38], which then determines downstream regulation of endogenous pro-hypertrophic pathways (WNT, IHH). Consistent with this model, we found that SMAD1/5/9 activation was strongly suppressed and undetectable in type X collagen-free chondral heparin–PEG explants [18] but only mildly and transiently reduced in vitro, where MSCs committed to endochondral development despite the presence of heparin. Unfortunately, we currently lack effective tools to specifically inhibit SMAD1/5/9, as MSC chondrogenesis is incompatible with genetic engineering approaches. Nonetheless, our study provides additional important support that SMAD1/5/9 activity plays a crucial role for instructing MSCs into the endochondral developmental lineage.

An important open question is why heparin used to retain TGF-β in a hydrogel can effectively instruct a chondral fate decision of MSCs in vivo but not in vitro. Since MSCs became hypertrophic in the heparin–PEG hydrogel in vitro even in the absence of exogenous soluble TGF-β, and reducing the TGF-β concentration to the minimal effective level in pellet cultures failed to prevent endochondral misdifferentiation, we believe that the additional supplementation of soluble TGF-β in the medium is unlikely to be the primary driver of the observed differentiation shift. Given that high sulfation was required for chondral fate instruction and partial desulfation lead to endochondral differentiation, it appears likely that sulfation-dependent binding of bioactive factors to heparin in vivo plays a prominent role. Indeed, heparin is known to bind a multitude of cytokines, including Gremlin [39], an enhancer of TGF-β-SMAD2/3 signaling and inhibitor of BMP-SMAD1/5/9 activation. Moreover, additional antihypertrophic signals in vivo are a likely possibility; PTHrP for example, can be produced by normal subcutaneous adipose tissue [40]. Furthermore, the well-recognized antiangiogenic activity of heparin [41] is also a likely contributor, as sufficient availability of calcium and blood supply are crucial for endochondral ossification and bone remodeling. Of note, here we showed that heparin reduced the endogenous production of PGE2 during MSC chondrogenesis, that we previously identified as an autocrine inhibitor of chondrocyte hypertrophy [31]. The standard chondrogenic medium contains high doses of the anti-inflammatory glucocorticoid dexamethasone that inhibits PGE2 synthesis [42]. Conversely, macrophages, endothelial cells, and mast cells are ready sources of PGE2 in the subcutaneous niche, and this may paracrinely contribute to suppress endochondral misdifferentiation of MSCs in heparin–PEG hydrogels in vivo. Altogether, while our data suggest extracellular SMAD1/5/9 inhibitors available in vivo and PGE2, further experiments are warranted to investigate the contribution of these and further potentially auxiliary bioactivities that aid TGF-β-loaded heparin–PEG hydrogels to instruct and stabilize the chondral lineage decision of MSCs in vivo.

One limitation of our study is that we did not directly compare in vitro and in vivo chondrogenesis in this study, but rather compared with our previous results. Naturally, batch-related variations in the molecular weight distribution and maleimide modification of heparin may have occurred between studies, and their impact on the heparin potency remains unclear. Still, we observed stable chondral development of a subcutaneous implant with the heparin–PEG batch used in the current study (data not shown). A similar point can be made for comparing heparin treatment with other antihypertrophic interventions, specifically WNT inhibition and pulsed PTHrP treatment. While we focused on their shared inability to fully instruct MSCs into chondral development, a direct comparison would provide a more comprehensive understanding of their relative effectiveness. Additionally, we did not examine endogenous heparan sulfates during in vitro chondrogenesis of MSCs or as hydrogel components. Instead, we intentionally selected heparin since it is already widely used in clinical settings, whereas the greater variability, diversity, and complexity of heparan sulfates may impede the potential clinical translation of these hydrogels for cartilage regeneration.

## Conclusions

In essence, we showed here for the first time that heparin dichotomously inhibited SMAD3 but not SMAD2 activation by TGF-β without affecting the chondrogenic differentiation of MSC cultures. Thus, as in the growth plate, SMAD2 appears to play a more prominent role than SMAD3 for in vitro chondrogenesis. We further propose that a stimulation of the insulin-induced AKT activation aids in maintaining phosphorylated SMAD2 levels. Moreover, we found that heparin exhibited antihypertrophic activity that was comparable in selectivity and effectivity with that of WNT inhibition and pulsed PTHrP treatment, previously considered the most effective interventions in that regard, but offered advantages with respect to cost, technical simplicity, and cell-compatible high-quality formulation. Most importantly, the in vitro effects of heparin fell short of its remarkable ability observed previously in vivo: Retaining TGF-β and permitting formation of permanent neocartilage fully devoid of type X collagen and exhibiting long-term resistance to endochondral ossification. While heparin appeared to sufficiently inhibit activation of the pro-hypertrophic WNT pathway, it had two notable limitations: incomplete silencing of SMAD1/5/9 activation and undesired inhibition of antihypertrophic PGE2. Thus, we here demonstrated that environmental contributions were essential to allow heparin–PEG to guide MSCs into the chondral instead of the endochondral lineage, and their identification may be key to successful stabilization of MSC chondrogenesis under fully defined in vitro conditions. Our ability to prevent endochondral ossification is crucial to pave the way for the safe and effective therapeutic use of MSCs for cartilage regeneration.

## Supplementary Information

Additional file 1 (Supplementary Figure S1. MSC in vitro chondrogenesis in heparin–PEG hydrogels versus pellet controls. MSCs were cultured as pellets (pellet ctrl; 5x10^5 cells) or in heparin–PEG hydrogels (H-PEG hydrogel; 1.2x10^6 cells) containing 22.4 mg/mL crosslinked heparin and 60 ng TGF-β1 for 4 weeks in chondrogenic medium with or without soluble TGF-β1 (10 ng/mL) in vitro. Microsections of day-28 samples were assessed via immunohistochemistry to detect type II collagen (scale bar: overview = 200 µm, magnification = 50 µm; n =1)).

Additional file 2 (Supplementary Figure S2. Representative controls corresponding to the immunohistochemical data shown in Figure 1A-B. Adequate previously characterized samples were recruited from earlier independent studies. All samples represent day 28 of MSC chondrogenesis performed either as pellet culture or in heparin–PEG hydrogels. Microsections were assessed via immunohistochemistry to detect either type II collagen, aggrecan, or type X collagen, as indicated (scale bar: overview = 200 µm, magnification = 50 µm)).

Additional file 3 (Supplementary Figure S3. Endochondral development of MSCs cultured either as pellets or in heparin–PEG hydrogels. In vitro culture of MSCs for 4 weeks in chondrogenic medium containing 10 µg/mL TGF β1 was performed either as pellets (pellet ctrl; 5×10^5 cells) or in heparin–PEG hydrogels (H-PEG hydrogel; 1.2×10^6 cells; 22.4 mg/mL crosslinked heparin, 120 ng TGF-β1). **A** ALP enzyme activity normalized to total protein levels was assessed in cell lysates of day-28 samples (n = 2). **B** Day-28 Ct values for the indicated reference genes were determined by qPCR. **C-D** Baseline (day 0) and day-28 gene expression levels of the indicated chondrocyte and hypertrophy markers, with *CPSF6* and *HPRT* used as reference genes. N = 4 experiments using independent MSC donor populations. Box plots were built as described in the statistics section. *p≤0.05 versus day 0, Mann-Whitney *U* test).

Additional file 4 (Supplementary Figure S4. MSC hypertrophic development under reduced or absent soluble TGF-β1. In vitro culture of MSCs for 4 weeks in chondrogenic medium with or without soluble TGF-β1, as indicated, was performed either as pellet culture (5×10^5 cells) or in heparin–PEG hydrogels (H-PEG hydrogel; 1.2×10^6 cells; 22.4 mg/mL crosslinked heparin, 120 ng TGF-β1). **A** Microsections of day-28 samples were assessed via immunohistochemistry to detect type II collagen or type X collagen (scale bar: overview = 200 µm, magnification = 50 µm). **B** Safranin O/Fast Green staining to assess proteoglycan accumulation; or immunohistochemical analysis for detection of type II collagen (scale bar: overview = 200 µm, magnification = 50 µm). **C** ALP enzyme activity was determined in the pooled supernatants of 4-5 MSC pellets at weekly intervals (n = 1)).

Additional file 5 (Supplementary Figure S5. All western blots included in Figure 2 quantifications. Detection of **A** phospho-SMAD2 and total SMAD2/3, **B** phospho-SMAD3 and total SMAD2/3, and **C** phospho-SMAD1/9 and total SMAD1/5 in whole protein lysates. β-actin served as an internal reference).

Additional file 6 (Supplementary Figure S6. Effect of soluble heparin on TGF-β-induced SMAD activation in MSCs in vitro. MSCs were cultured as pellets for 4 weeks in standard chondrogenic medium supplemented with soluble heparin (0, 10, 100, 700 µg/mL). On day 28, serum-free, defined chondrogenic medium, with or without TGF-β1 (10 ng/mL), was pre-incubated with soluble heparin (0, 10, 100, 700 µg/mL) for 60 minutes and then added to the MSC pellets. TGF-β1-free controls had received TGF-β1 for 28 days, followed by a 30-minute TGF-β1 starvation. After 30 minutes, cells were harvested and whole protein lysates were prepared for western blotting. **A** Phospho-SMAD2 and total SMAD2/3, **B** phospho-SMAD3 and total SMAD2/3, or **C** phospho-SMAD1/9 and total SMAD1/5 were detected. β-actin was used as an additional internal reference. All samples in each line were run on the same gel and blotted onto one membrane. Samples from 2 independent donors are shown).

Additional file 7 (Supplementary Figure S7. All western blots included in Figure 3 quantifications. **A** Whole protein lysates were used to detect phospho-AKT and total AKT. **B** β-catenin was assessed in membrane-depleted cell extracts. β-actin was used as an internal reference. The transmembrane protein CD81 was used to confirm the successful depletion of the membrane fraction).

Additional file 8 (Supplementary Figure S8. MSC in vitro chondrogenesis in the presence of soluble heparin. MSCs were cultured as pellets for 4 weeks in standard chondrogenic medium (including 10 ng/mL TGF-β1 and 6.25 µg/mL insulin) that was supplemented with soluble heparin (0, 10, 100, 700 µg/mL). **A-C** Gene expression levels of chondrocyte markers as designated, using *CPSF6* and *HPRT* as reference genes (n = 5). **D** Day 28 cycle threshold (Ct) values for the indicated reference genes were assessed by qPCR. **E** DNA content per pellet was assessed using PicoGreen fluorescent probe. **F** Differentiating MSCs on day 28 were analyzed for PGE2 secretion levels using ELISA. **G** The PGE2 standard solution from the PGE2 immunoassay kit was supplemented with 700 µg/mL soluble heparin. PGE2 levels were assessed via spectrophotometry (n = 1). Box plots were built as described in the statistics section with a dashed line representing control samples set to 1. *p≤0.05 versus day 28 control samples, Mann-Whitney *U* test).

Additional file 9 (Supplementary Figure S9. Hypertrophic development of MSC-derived chondrocytes in presence of soluble heparin. MSCs were cultured as pellets for 4 weeks in standard chondrogenic medium supplemented with soluble heparin (0 and 10 µg/mL). **A** Gene expression levels of hypertrophy markers as designated, using *CPSF6* and *HPRT* as reference genes (n = 5). **B** Expression levels of genes encoding ALK receptors as designated, using *CPSF6* and *HPRT* as reference genes (n = 5). **C** Heparin (0, 10, 100, 700 µg/mL) was added to MSC-conditioned positive control supernatants as specified. ALP enzyme activity in the culture supernatants was assessed spectrophotometrically via substrate conversion (n = 2). Box plots were built as described in the statistics section. Extreme outliers according to Tukey’s Fences test are indicated with empty circles. *p≤0.05 versus day 28 control samples, Mann-Whitney *U* test).

Additional file 10 (Supplementary Figure S10. Western blot analysis of type X collagen in all MSC donor populations included in Figure 5. Type II collagen was used as an internal reference (uncropped blot pictures are provided as Supplementary Material S1)).

Additional file 11.

Additional file 12 (Supplementary Table S1. Alphabetical list of primer sequences utilized for qPCR analysis).

## Data Availability

The authors confirm unrestricted access to all raw data, statistical analysis, and materials used in this study upon reasonable request.

## Abbreviations

ACAN: Aggrecan
ALP: Alkaline phosphatase
COL2A1: Collagen type II alpha 1 chain
COL10A1: Collagen type X alpha 1 chain
CPSF6: Cleavage and polyadenylation specific factor 6
GAPDH: Glyceraldehyde-3-phosphate dehydrogenase
GDF5: Growth and differentiation factor 5
ELISA: Enzyme-linked immunosorbent assay
GLI1: GLI family zinc finger 1
H–PEG: Heparin–PEG hydrogel
HPRT: Hypoxanthine phosphoribosyltransferase
IBSP: Integrin-binding sialoprotein
IHH: Indian Hedgehog
IQR: Interquartile range
MEF2C: Myocyte enhancer factor 2C
MMP: Matrix metallopeptidase
mRNA: Messenger ribonucleic acid
MSC: Mesenchymal stromal cell
PEG: Poly(ethylene glycol)
PGE2: Prostaglandin E2
PTHrP: Parathyroid hormone-related protein
qPCR: Quantitative polymerase chain reaction
sGAGs: Sulfated glycosaminoglycans
SMAD: Small mother against decapentaplegic
SDS: Sodium dodecyl sulfate
SOX9: SRY-box transcription factor 9
TGF-β: Transforming growth factor beta
WNT: Wingless-int

## Declarations

### Declaration of AI and AI-assisted technologies in the writing process

During the preparation of this work, the authors used YoKI (meta-llama/Meta-Llama-3.1-70B) to assist with language editing and Perplexity (https://www.perplexity.ai) for literature research. After using these tools, the authors reviewed and edited the content as needed and take full responsibility for the content of the publication.
