# Supplementary figures and images for: Dichotomous SMAD2/3 regulation and selective antihypertrophic activity of heparin during in vitro chondrogenesis of mesenchymal stromal cells
Source: Cell Mol Biol Lett. 2026 Mar 17;31:51. doi: 10.1186/s11658-026-00899-8 (PMC13064404; doi:10.1186/s11658-026-00899-8)

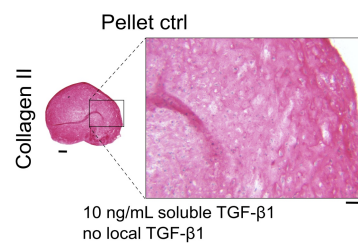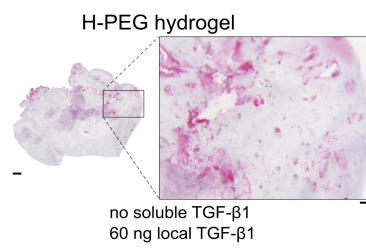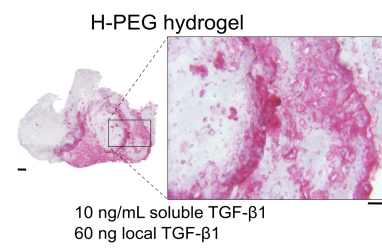

Supplement: Supplementary file 1 — Additional file 1 (Supplementary Figure S1. MSC in vitro chondrogenesis in heparin–PEG hydrogels versus pellet controls. MSCs were cultured as pellets (pellet ctrl; 5x10^5 cells) or in heparin–PEG hydrogels (H-PEG hydrogel; 1.2x10^6 cells) containing 22.4 mg/mL crosslinked heparin and 60 ng TGF-β1 for 4 weeks in chondrogenic medium with or without soluble TGF-β1 (10 ng/mL) in vitro. Microsections of day-28 samples were assessed via immunohistochemistry to detect type II collagen (scale bar: overview = 200 µm, magnification = 50 µm; n =1)). [file 11658_2026_899_MOESM1_ESM.pdf]

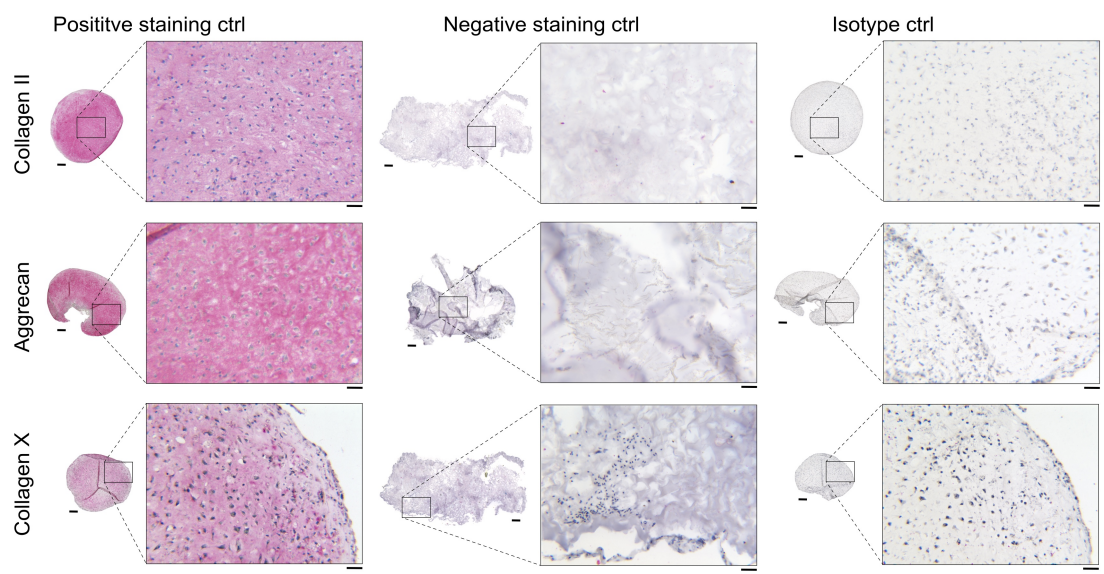

Supplement: Supplementary file 2 — Additional file 2 (Supplementary Figure S2. Representative controls corresponding to the immunohistochemical data shown in Figure 1A-B. Adequate previously characterized samples were recruited from earlier independent studies. All samples represent day 28 of MSC chondrogenesis performed either as pellet culture or in heparin–PEG hydrogels. Microsections were assessed via immunohistochemistry to detect either type II collagen, aggrecan, or type X collagen, as indicated (scale bar: overview = 200 µm, magnification = 50 µm)). [file 11658_2026_899_MOESM2_ESM.pdf]

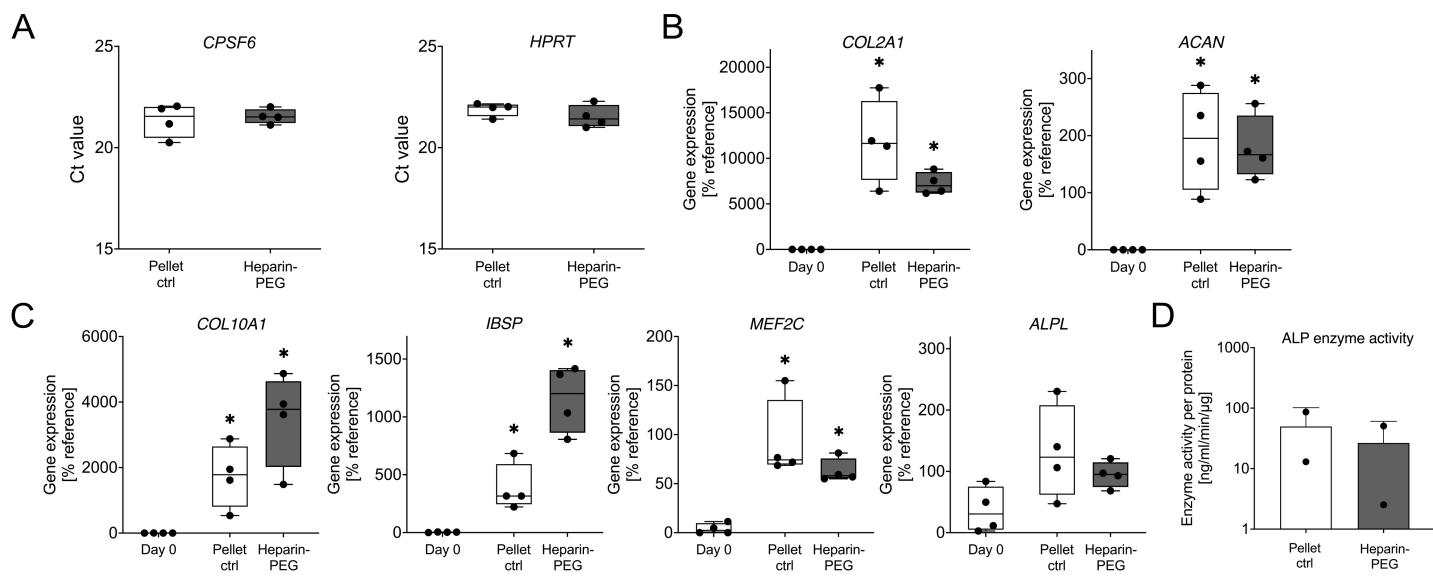

Supplement: Supplementary file 3 — Additional file 3 (Supplementary Figure S3. Endochondral development of MSCs cultured either as pellets or in heparin–PEG hydrogels. In vitro culture of MSCs for 4 weeks in chondrogenic medium containing 10 µg/mL TGF β1 was performed either as pellets (pellet ctrl; 5×10^5 cells) or in heparin–PEG hydrogels (H-PEG hydrogel; 1.2×10^6 cells; 22.4 mg/mL crosslinked heparin, 120 ng TGF-β1). A ALP enzyme activity normalized to total protein levels was assessed in cell lysates of day-28 samples (n = 2). B Day-28 Ct values for the indicated reference genes were determined by qPCR. C-D Baseline (day 0) and day-28 gene expression levels of the indicated chondrocyte and hypertrophy markers, with CPSF6 and HPRT used as reference genes. N = 4 experiments using independent MSC donor populations. Box plots were built as described in the statistics section. *p≤0.05 versus day 0, Mann-Whitney U test). [file 11658_2026_899_MOESM3_ESM.pdf]

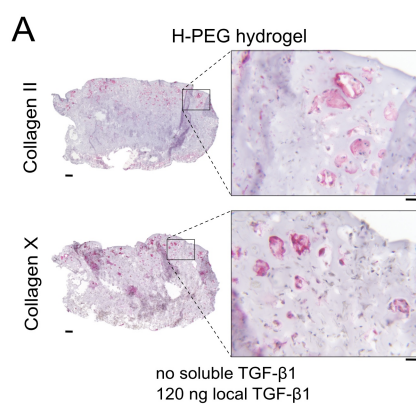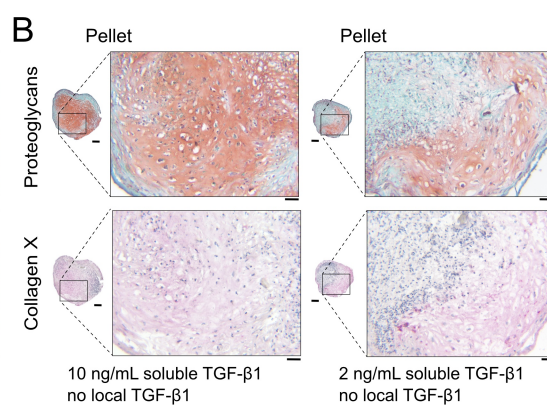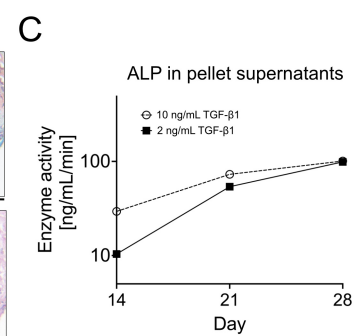

Supplement: Supplementary file 4 — Additional file 4 (Supplementary Figure S4. MSC hypertrophic development under reduced or absent soluble TGF-β1. In vitro culture of MSCs for 4 weeks in chondrogenic medium with or without soluble TGF-β1, as indicated, was performed either as pellet culture (5×10^5 cells) or in heparin–PEG hydrogels (H-PEG hydrogel; 1.2×10^6 cells; 22.4 mg/mL crosslinked heparin, 120 ng TGF-β1). A Microsections of day-28 samples were assessed via immunohistochemistry to detect type II collagen or type X collagen (scale bar: overview = 200 µm, magnification = 50 µm). B Safranin O/Fast Green staining to assess proteoglycan accumulation; or immunohistochemical analysis for detection of type II collagen (scale bar: overview = 200 µm, magnification = 50 µm). C ALP enzyme activity was determined in the pooled supernatants of 4-5 MSC pellets at weekly intervals (n = 1)). [file 11658_2026_899_MOESM4_ESM.pdf]

Pellet

A

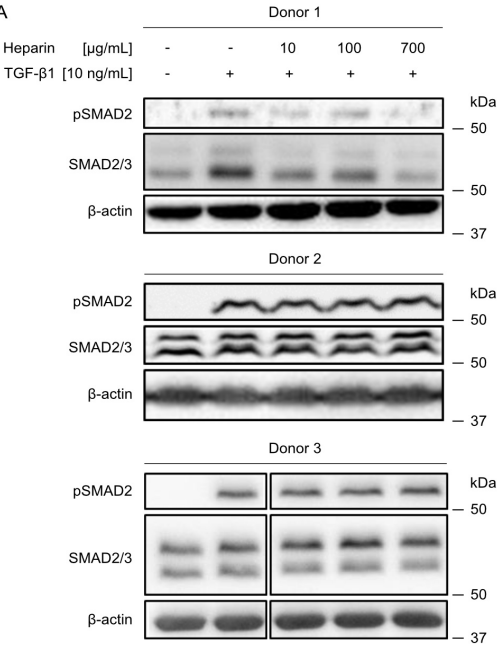

B

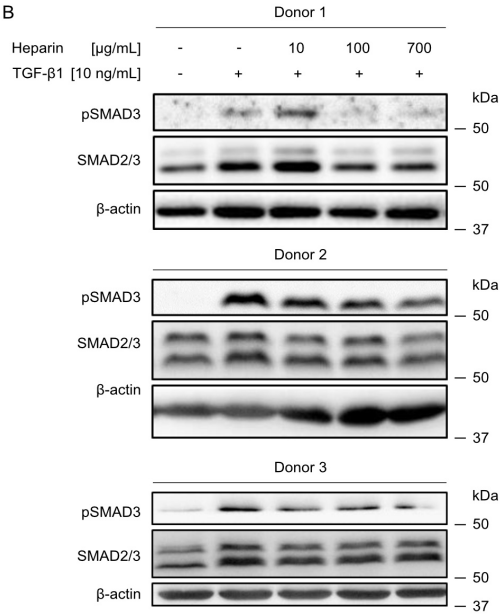

C

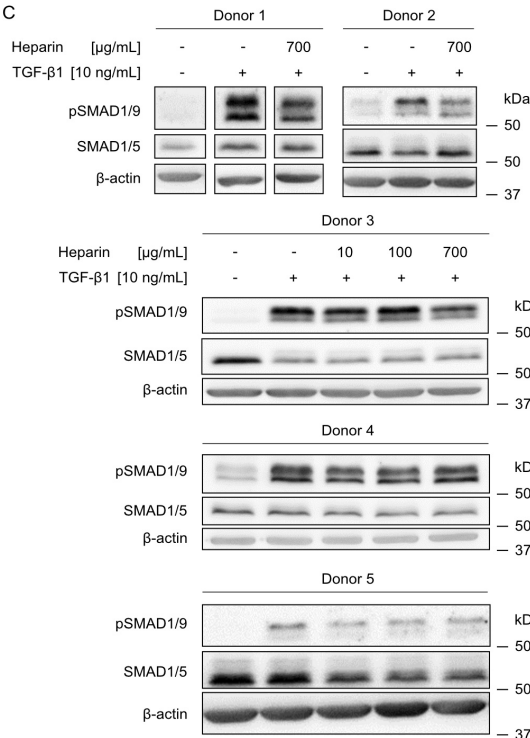

Supplement: Supplementary file 5 — Additional file 5 (Supplementary Figure S5. All western blots included in Figure 2 quantifications. Detection of A phospho-SMAD2 and total SMAD2/3, B phospho-SMAD3 and total SMAD2/3, and C phospho-SMAD1/9 and total SMAD1/5 in whole protein lysates. β-actin served as an internal reference). [file 11658_2026_899_MOESM5_ESM.pdf]

## Pellet

A

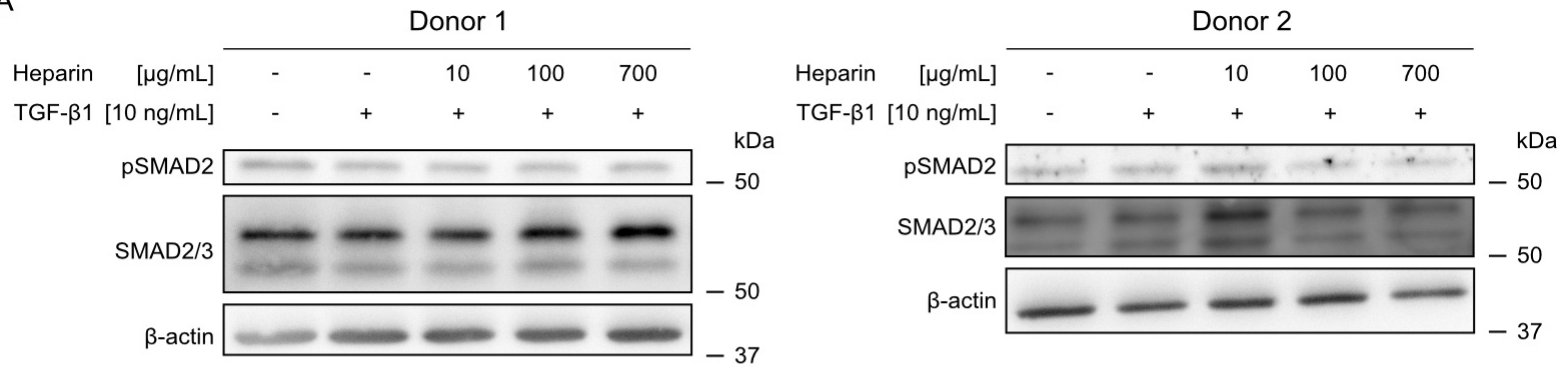

B

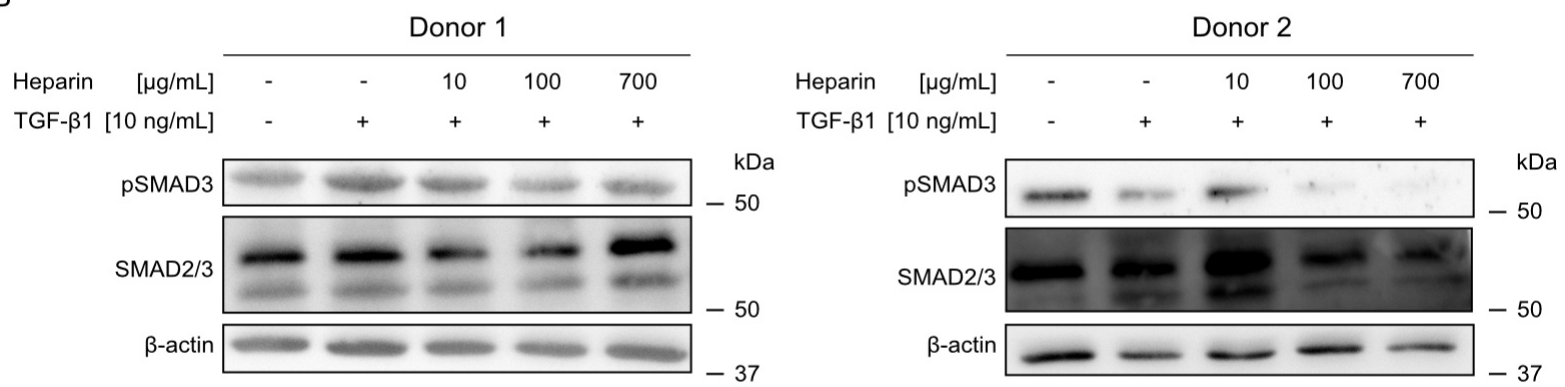

C

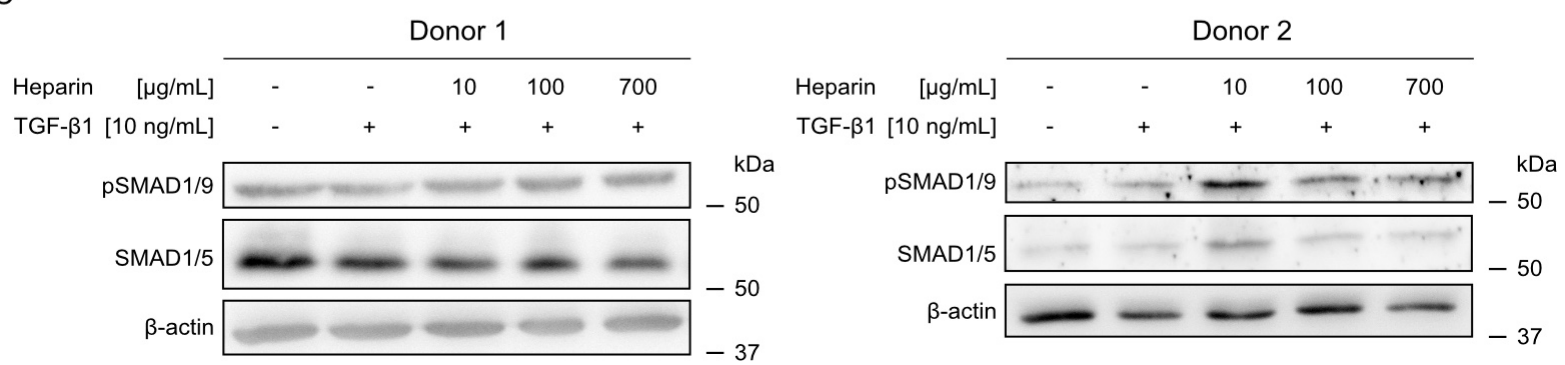

Supplement: Supplementary file 6 — Additional file 6 (Supplementary Figure S6. Effect of soluble heparin on TGF-β-induced SMAD activation in MSCs in vitro. MSCs were cultured as pellets for 4 weeks in standard chondrogenic medium supplemented with soluble heparin (0, 10, 100, 700 µg/mL). On day 28, serum-free, defined chondrogenic medium, with or without TGF-β1 (10 ng/mL), was pre-incubated with soluble heparin (0, 10, 100, 700 µg/mL) for 60 minutes and then added to the MSC pellets. TGF-β1-free controls had received TGF-β1 for 28 days, followed by a 30-minute TGF-β1 starvation. After 30 minutes, cells were harvested and whole protein lysates were prepared for western blotting. A Phospho-SMAD2 and total SMAD2/3, B phospho-SMAD3 and total SMAD2/3, or C phospho-SMAD1/9 and total SMAD1/5 were detected. β-actin was used as an additional internal reference. All samples in each line were run on the same gel and blotted onto one membrane. Samples from 2 independent donors are shown). [file 11658_2026_899_MOESM6_ESM.pdf]

A

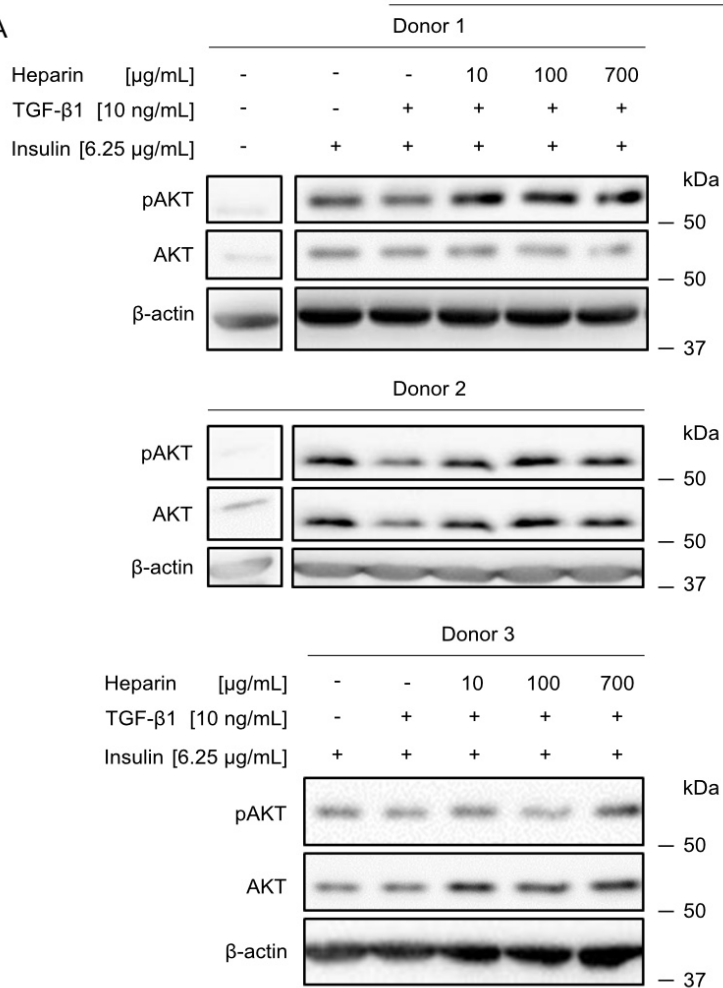

B

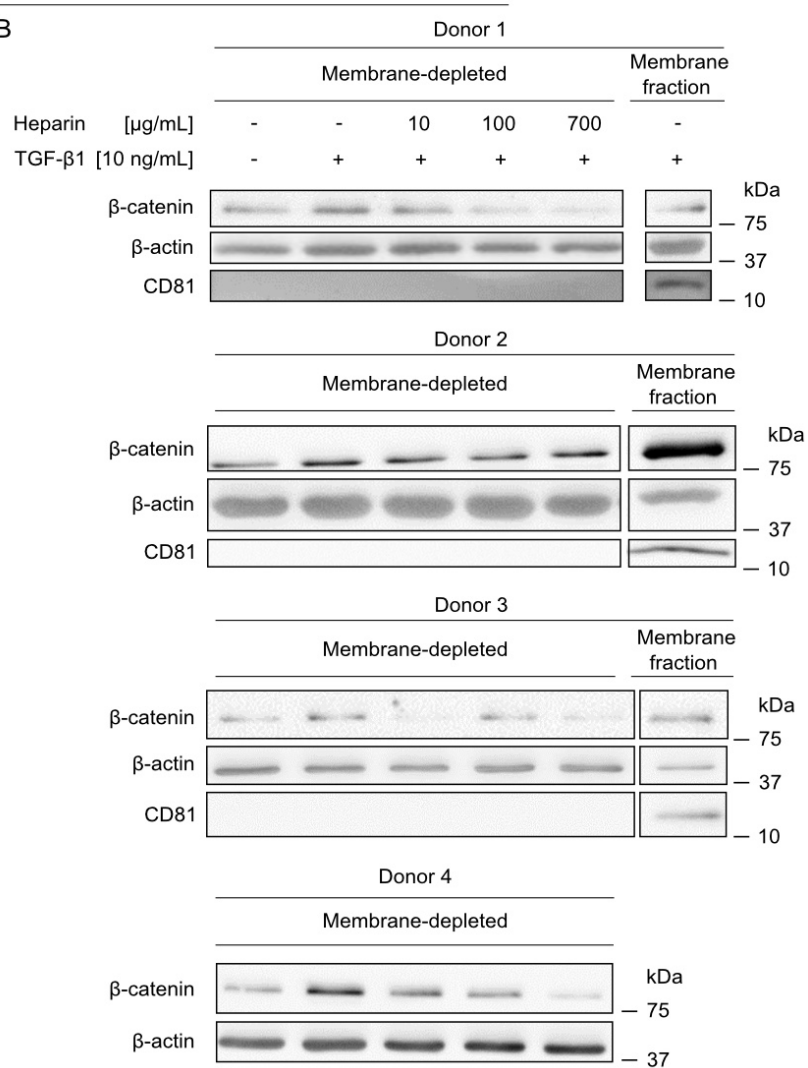

Supplement: Supplementary file 7 — Additional file 7 (Supplementary Figure S7. All western blots included in Figure 3 quantifications. A Whole protein lysates were used to detect phospho-AKT and total AKT. B β-catenin was assessed in membrane-depleted cell extracts. β-actin was used as an internal reference. The transmembrane protein CD81 was used to confirm the successful depletion of the membrane fraction). [file 11658_2026_899_MOESM7_ESM.pdf]

# Pellet

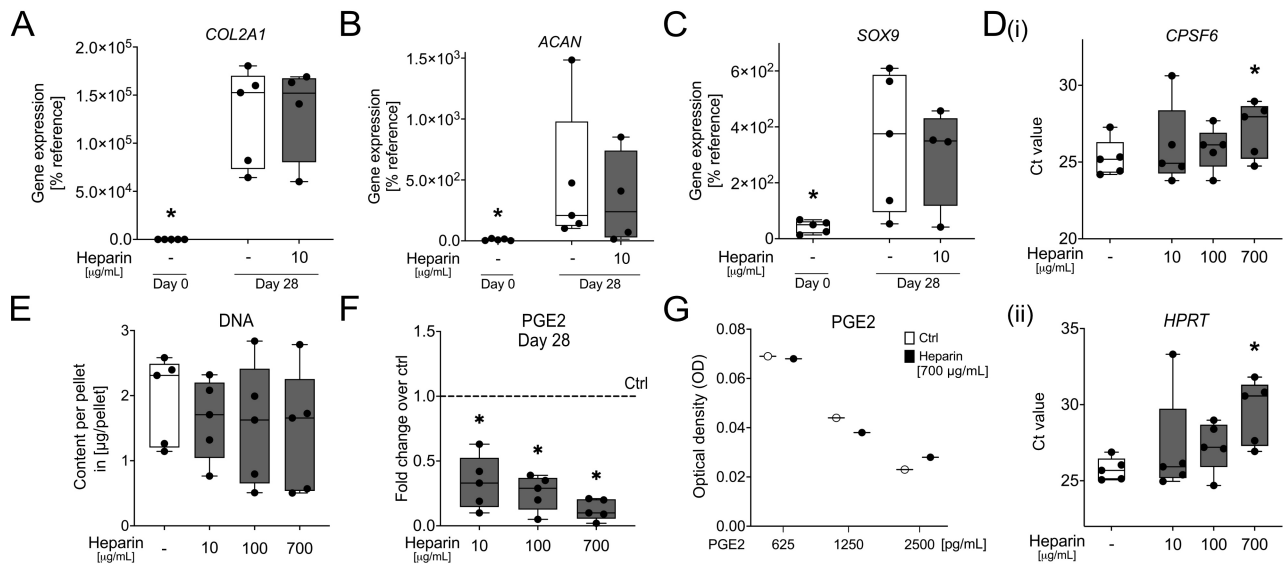

Supplement: Supplementary file 8 — Additional file 8 (Supplementary Figure S8. MSC in vitro chondrogenesis in the presence of soluble heparin. MSCs were cultured as pellets for 4 weeks in standard chondrogenic medium (including 10 ng/mL TGF-β1 and 6.25 µg/mL insulin) that was supplemented with soluble heparin (0, 10, 100, 700 µg/mL). A-C Gene expression levels of chondrocyte markers as designated, using CPSF6 and HPRT as reference genes (n = 5). D Day 28 cycle threshold (Ct) values for the indicated reference genes were assessed by qPCR. E DNA content per pellet was assessed using PicoGreen fluorescent probe. F Differentiating MSCs on day 28 were analyzed for PGE2 secretion levels using ELISA. G The PGE2 standard solution from the PGE2 immunoassay kit was supplemented with 700 µg/mL soluble heparin. PGE2 levels were assessed via spectrophotometry (n = 1). Box plots were built as described in the statistics section with a dashed line representing control samples set to 1. *p≤0.05 versus day 28 control samples, Mann-Whitney U test). [file 11658_2026_899_MOESM8_ESM.pdf]

# Pellet

A

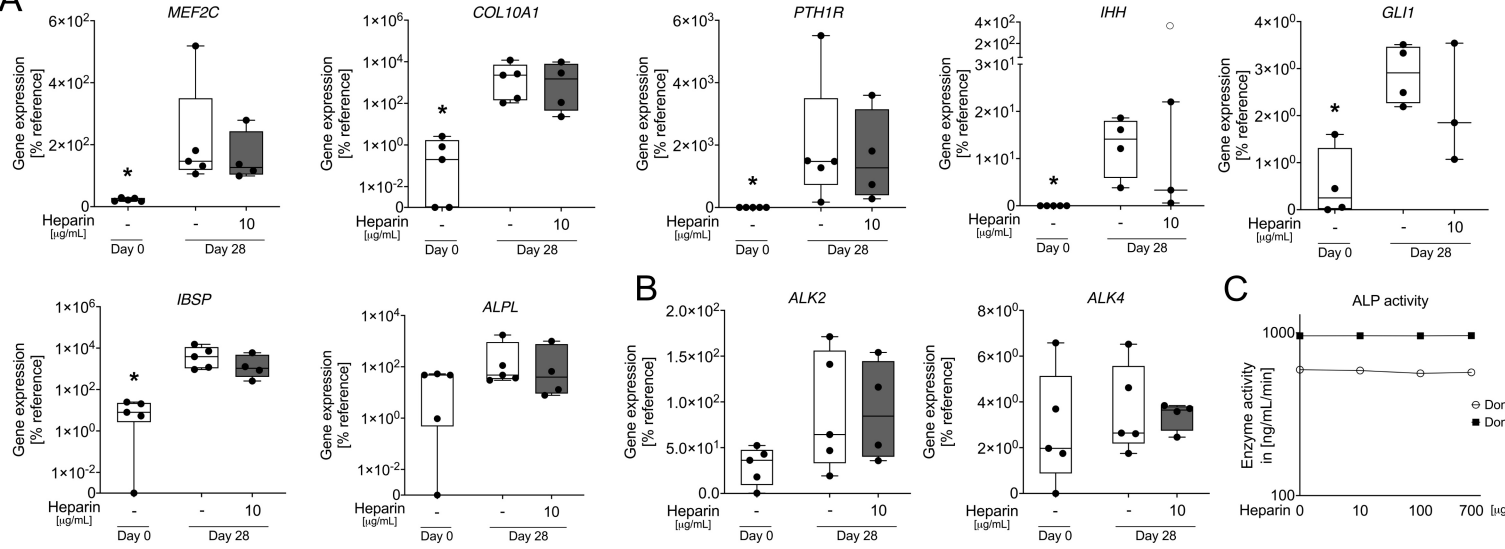

B

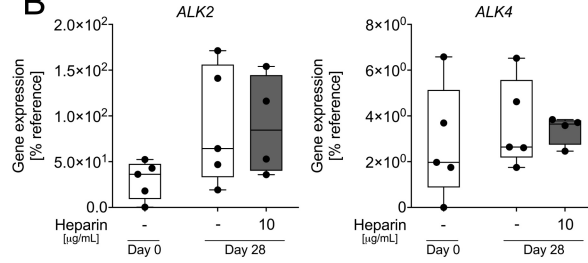

C

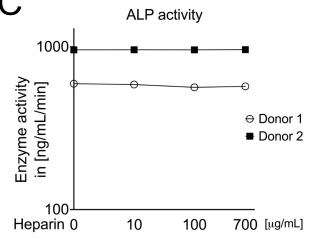

Supplement: Supplementary file 9 — Additional file 9 (Supplementary Figure S9. Hypertrophic development of MSC-derived chondrocytes in presence of soluble heparin. MSCs were cultured as pellets for 4 weeks in standard chondrogenic medium supplemented with soluble heparin (0 and 10 µg/mL). A Gene expression levels of hypertrophy markers as designated, using CPSF6 and HPRT as reference genes (n = 5). B Expression levels of genes encoding ALK receptors as designated, using CPSF6 and HPRT as reference genes (n = 5). C Heparin (0, 10, 100, 700 µg/mL) was added to MSC-conditioned positive control supernatants as specified. ALP enzyme activity in the culture supernatants was assessed spectrophotometrically via substrate conversion (n = 2). Box plots were built as described in the statistics section. Extreme outliers according to Tukey’s Fences test are indicated with empty circles. *p≤0.05 versus day 28 control samples, Mann-Whitney U test). [file 11658_2026_899_MOESM9_ESM.pdf]

Pellet

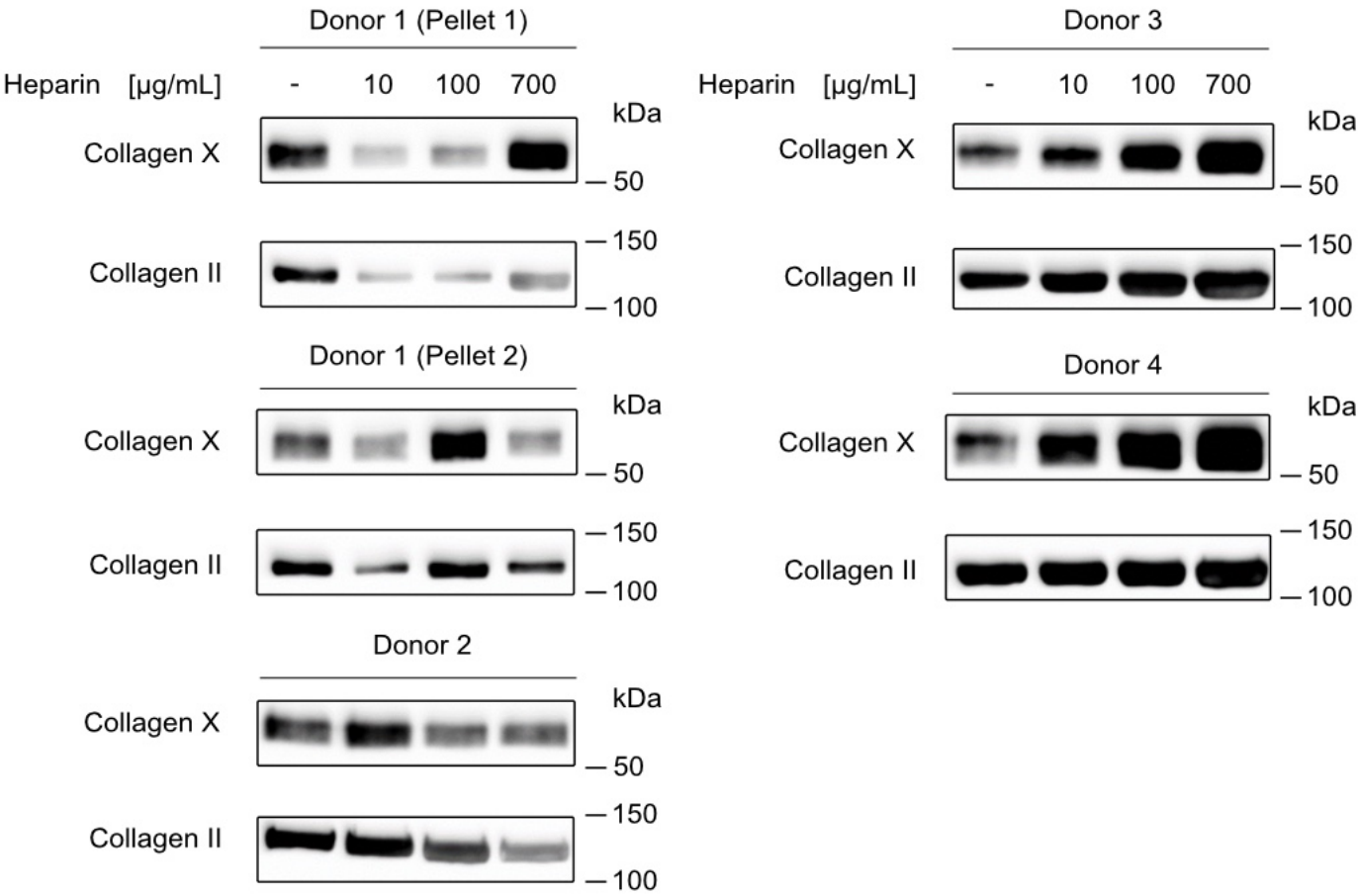

Supplement: Supplementary file 10 — Additional file 10 (Supplementary Figure S10. Western blot analysis of type X collagen in all MSC donor populations included in Figure 5. Type II collagen was used as an internal reference (uncropped blot pictures are provided as Supplementary Material S1)). [file 11658_2026_899_MOESM10_ESM.pdf]
