# Supplementary material for: Dichotomous SMAD2/3 regulation and selective antihypertrophic activity of heparin during in vitro chondrogenesis of mesenchymal stromal cells
Source: Cell Mol Biol Lett. 2026 Mar 17;31:51. doi: 10.1186/s11658-026-00899-8 (PMC13064404; doi:10.1186/s11658-026-00899-8)
Supplement: Supplementary file 11 — Additional file 11. [file 11658_2026_899_MOESM11_ESM.pdf]

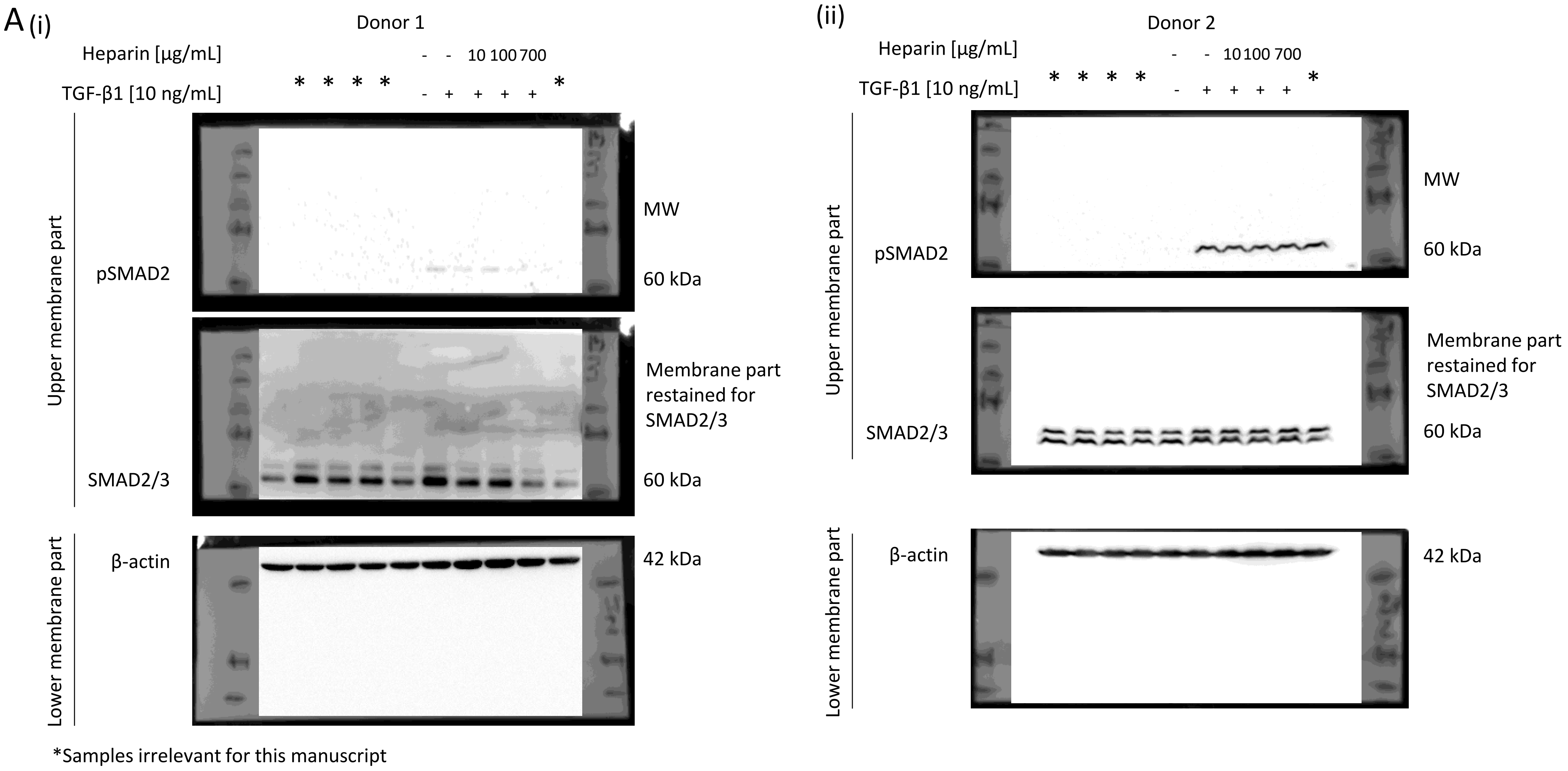

Supplementary Material S1. Western blots included in this study shown as full uncropped images.

(iii)

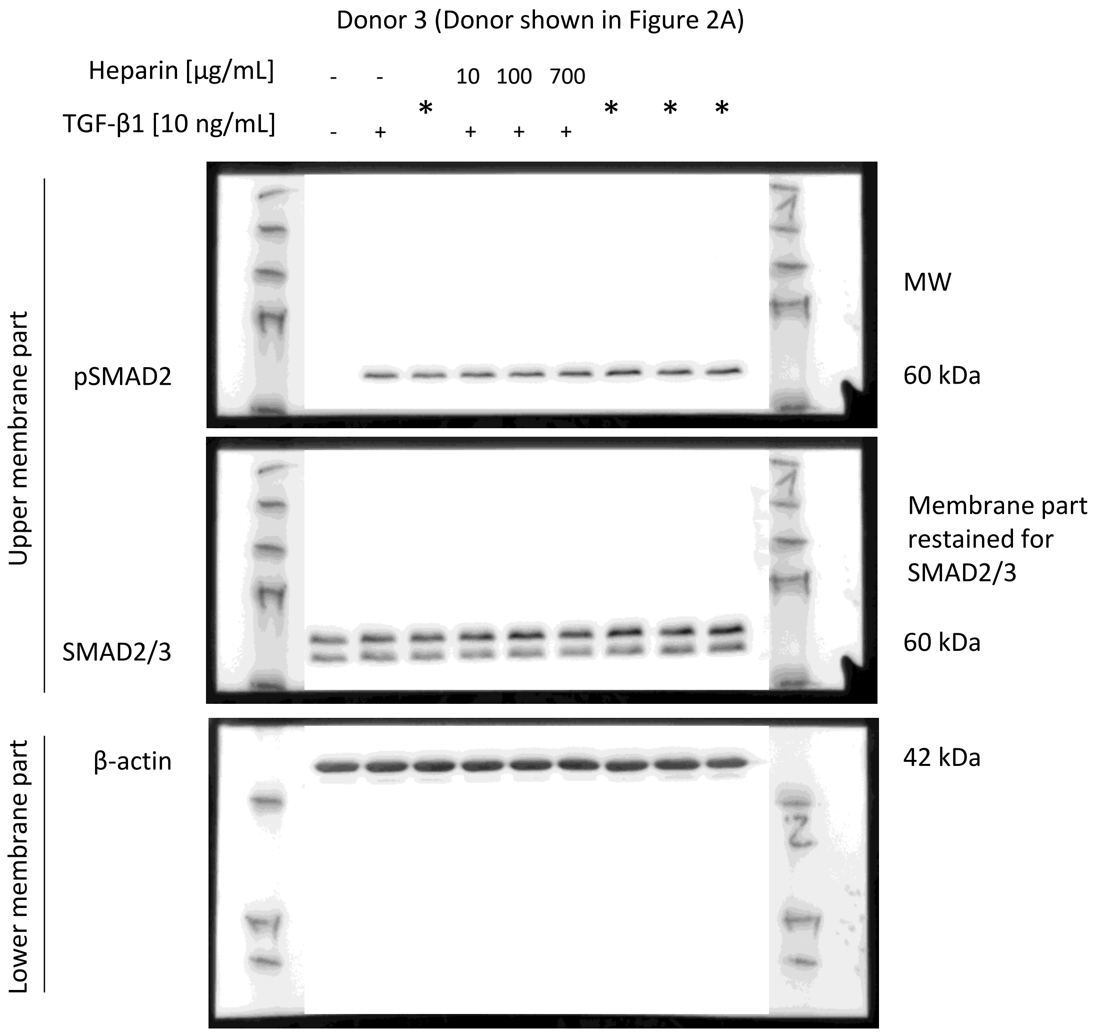

*Samples irrelevant for this manuscript

Supplementary Material S1. Western blots included in this study shown as full uncropped images.

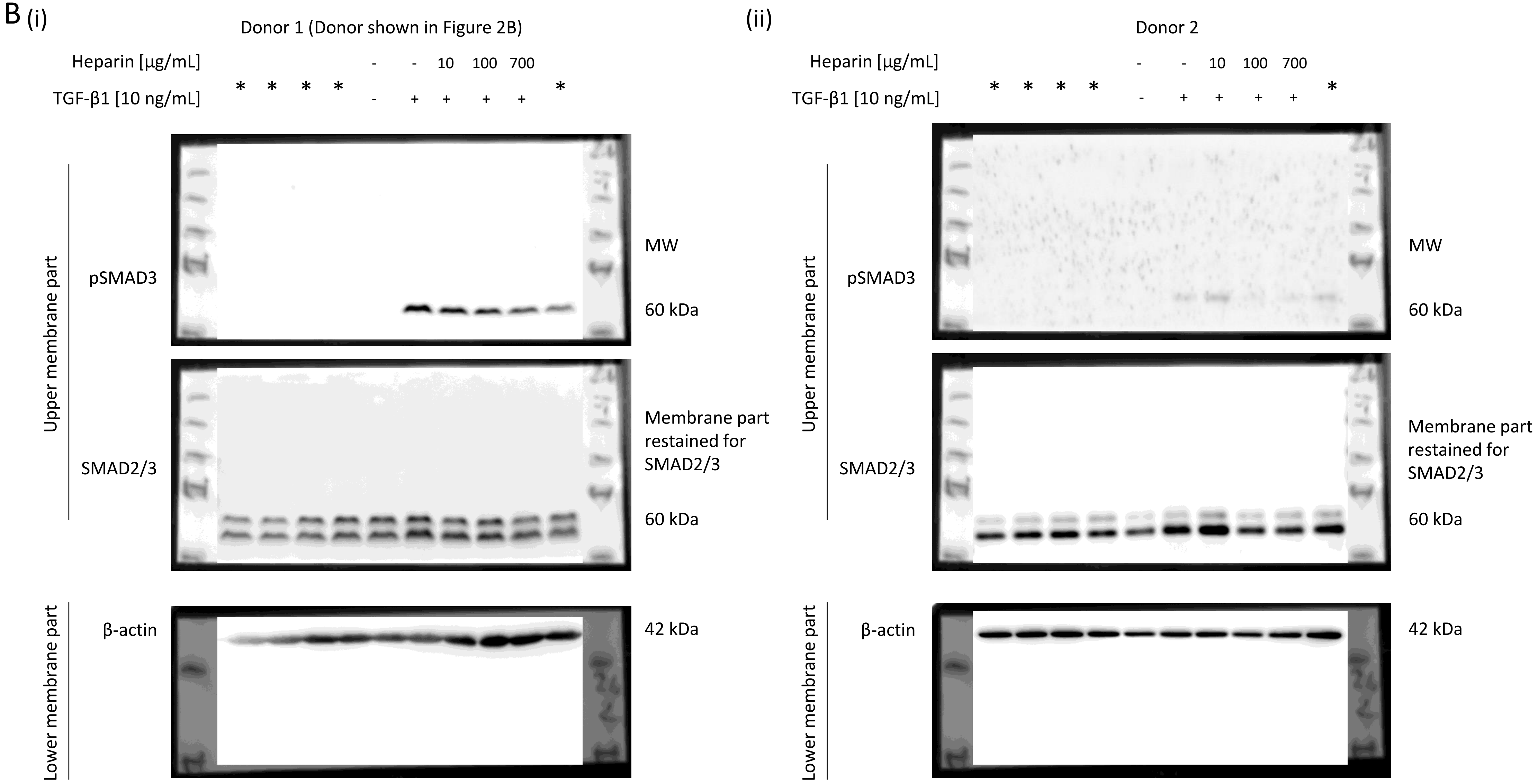

*Samples irrelevant for this manuscript

Supplementary Material S1. Western blots included in this study shown as full uncropped images.

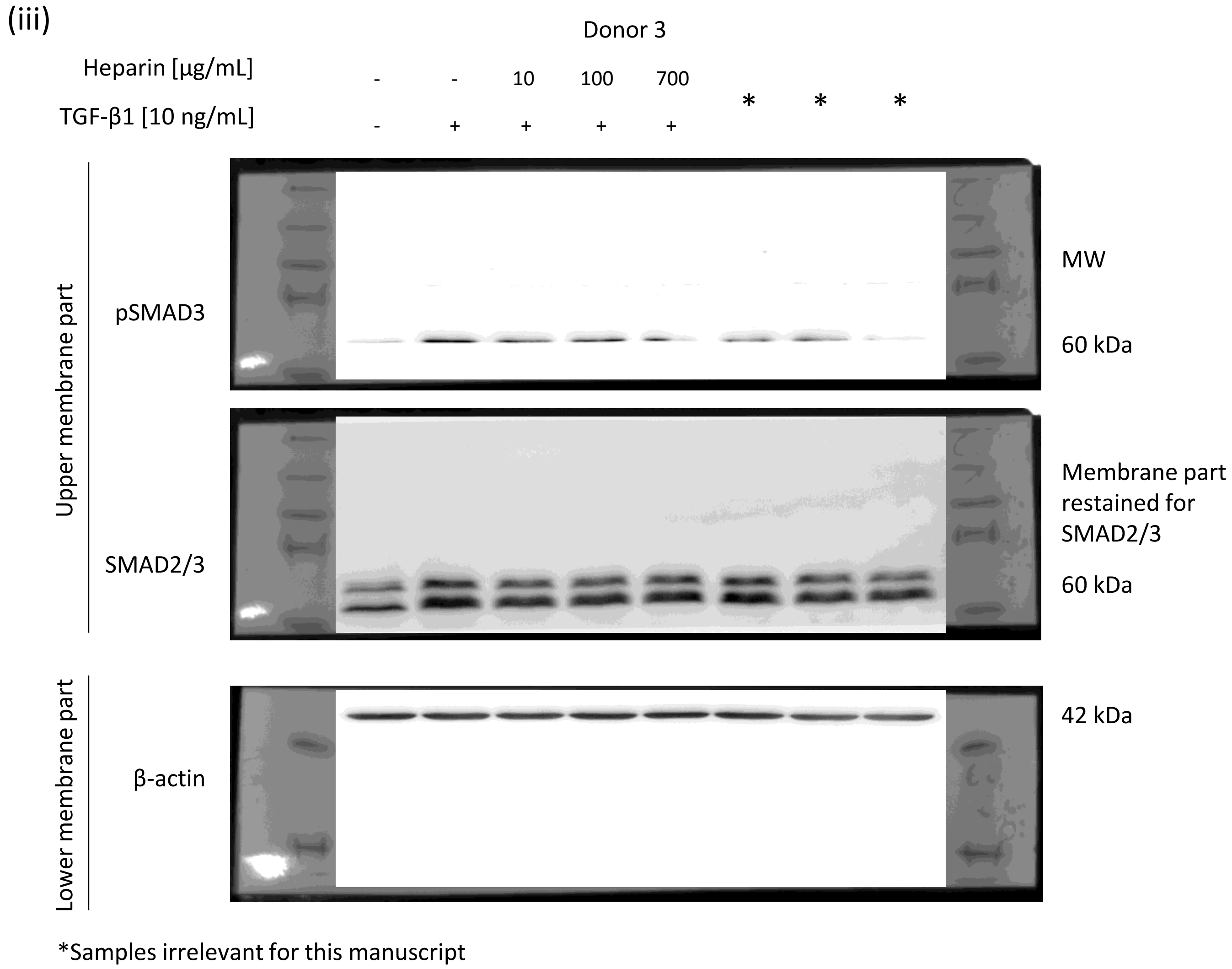

Supplementary Material S1. Western blots included in this study shown as full uncropped images.

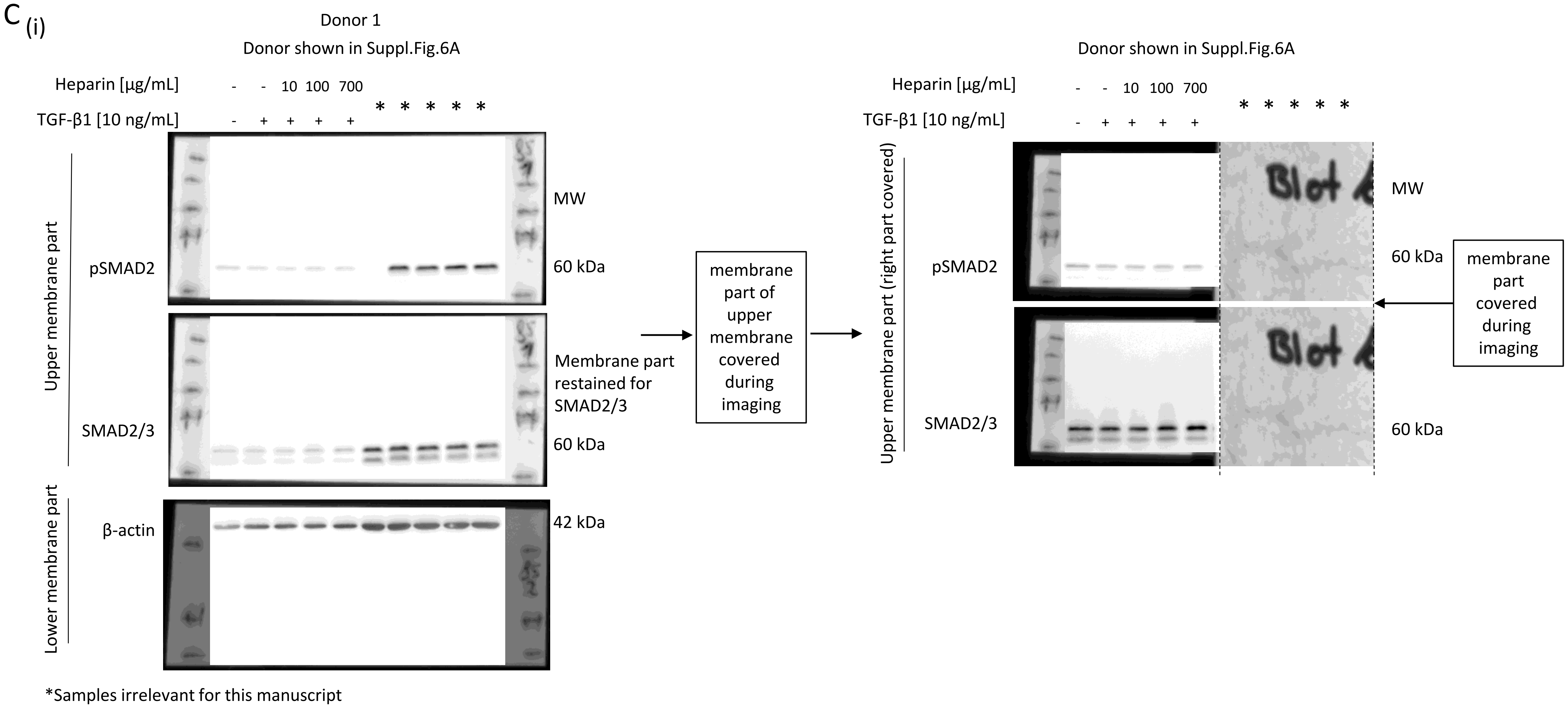

Supplementary Material S1. Western blots included in this study shown as full uncropped images.

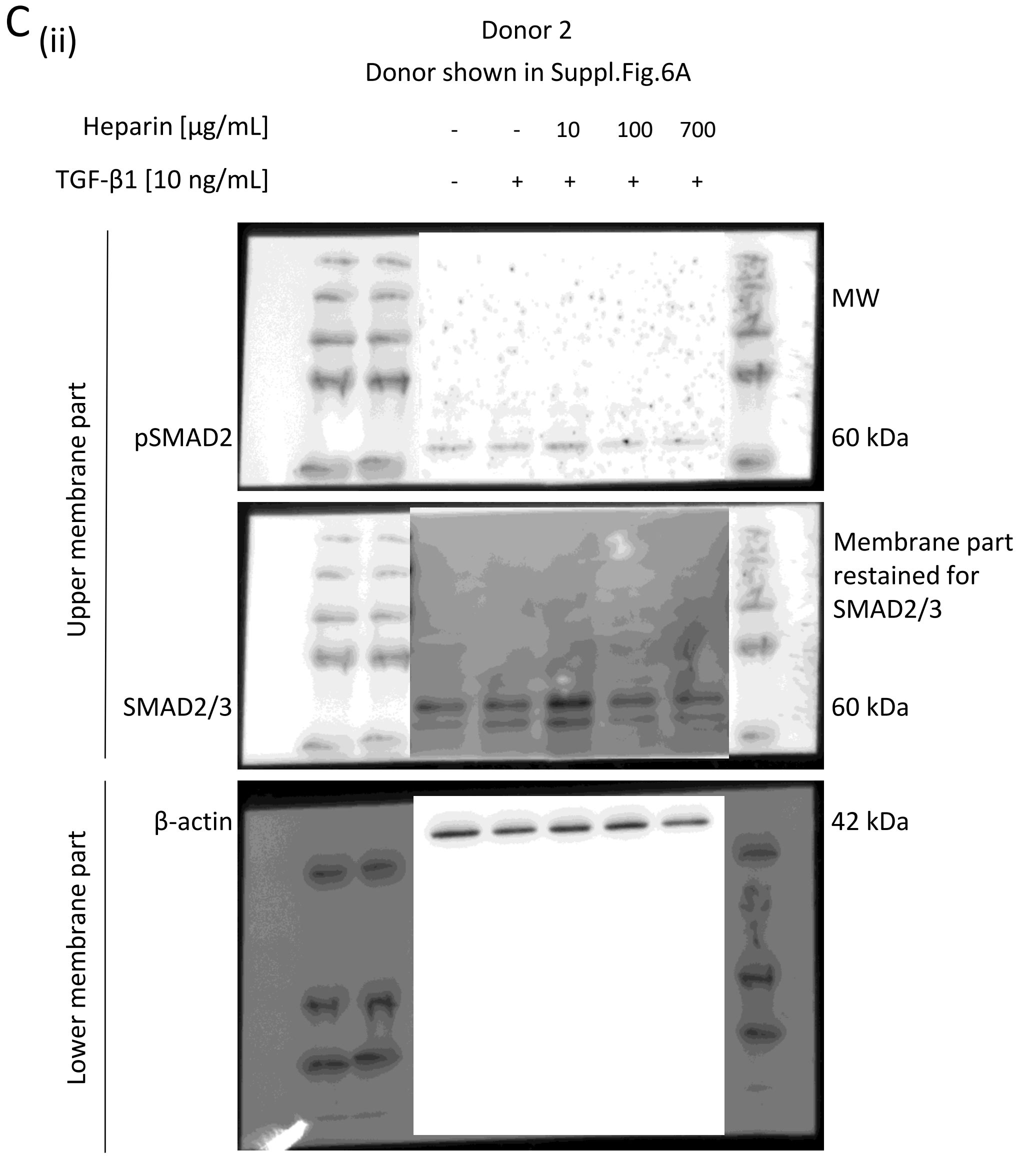

Supplementary Material S1. Western blots included in this study shown as full uncropped images.

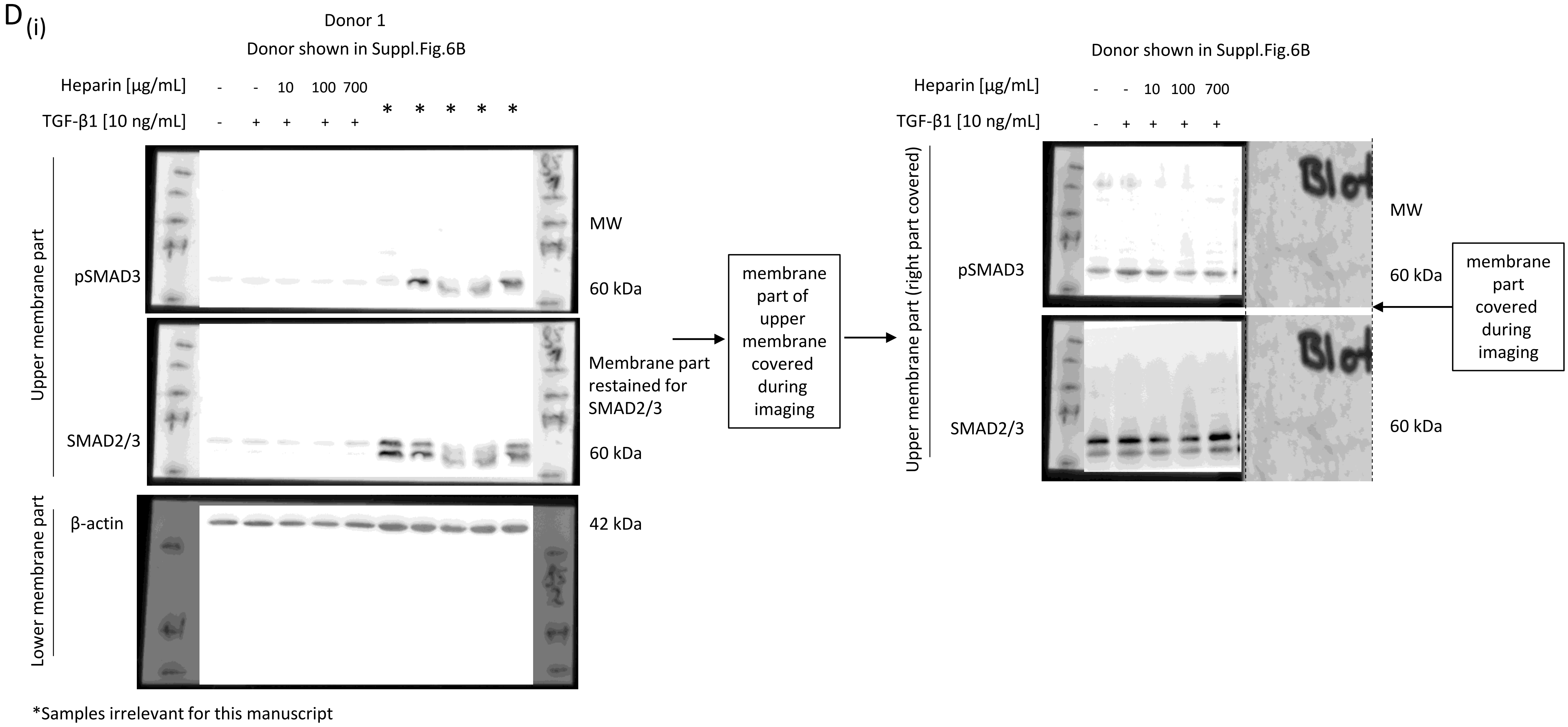

Supplementary Material S1. Western blots included in this study shown as full uncropped images.

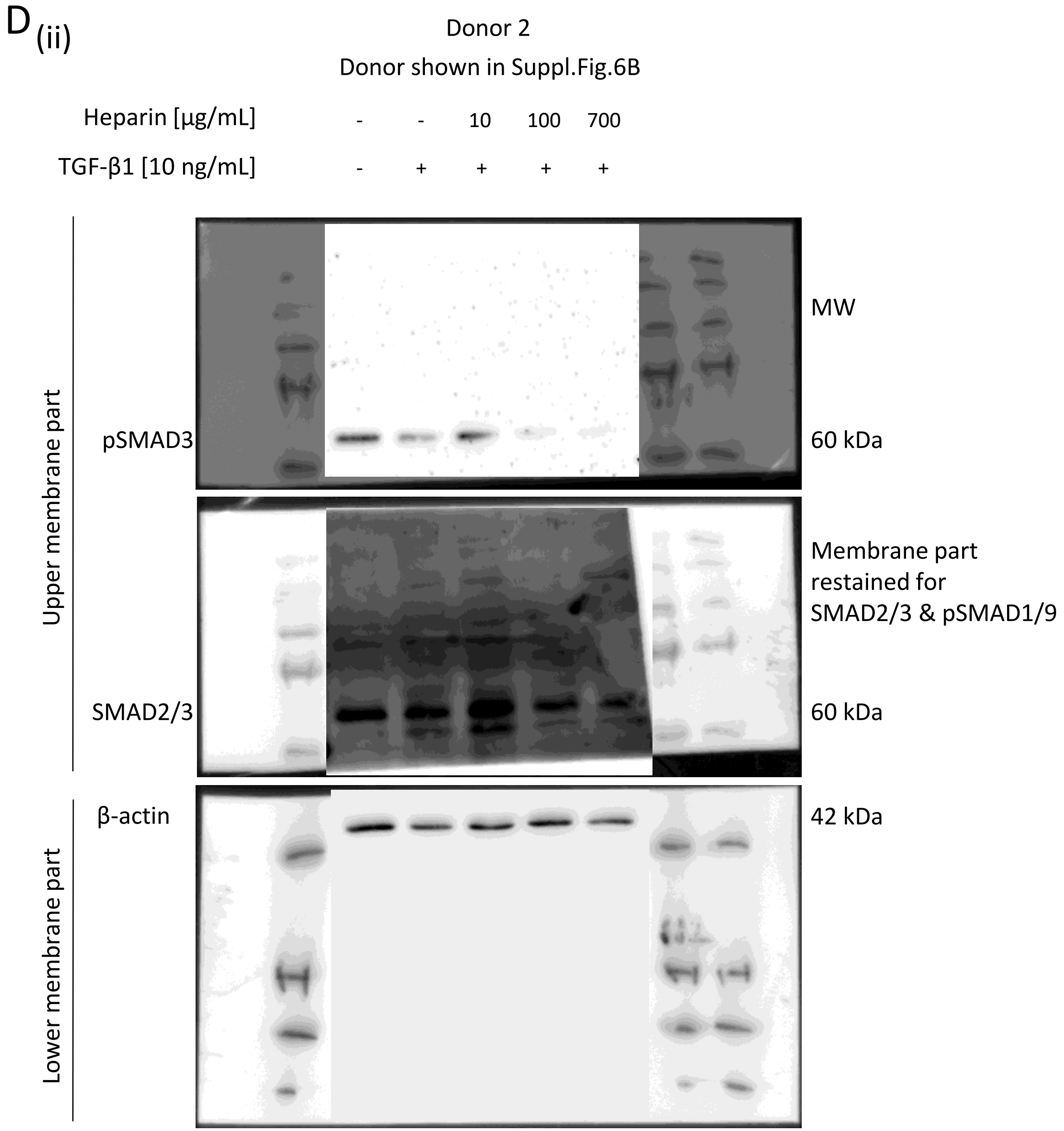

Supplementary Material S1. Western blots included in this study shown as full uncropped images.

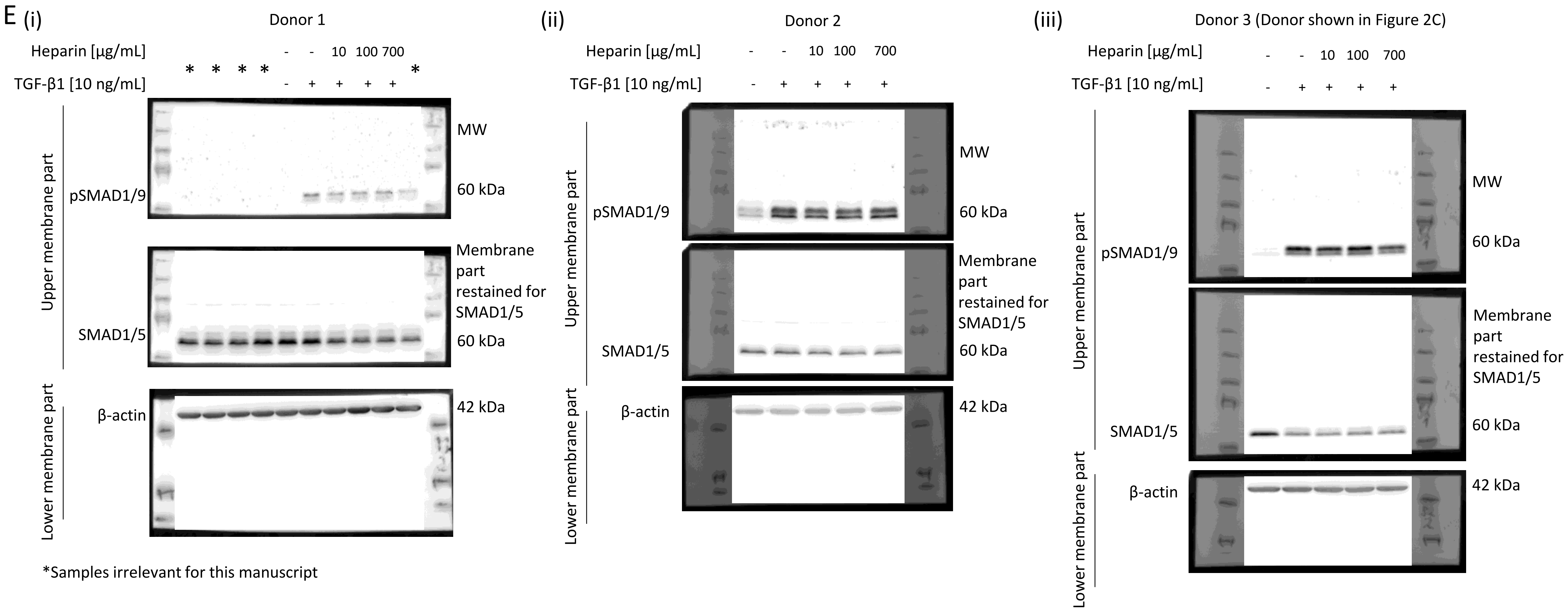

Supplementary Material S1. Western blots included in this study shown as full uncropped images.

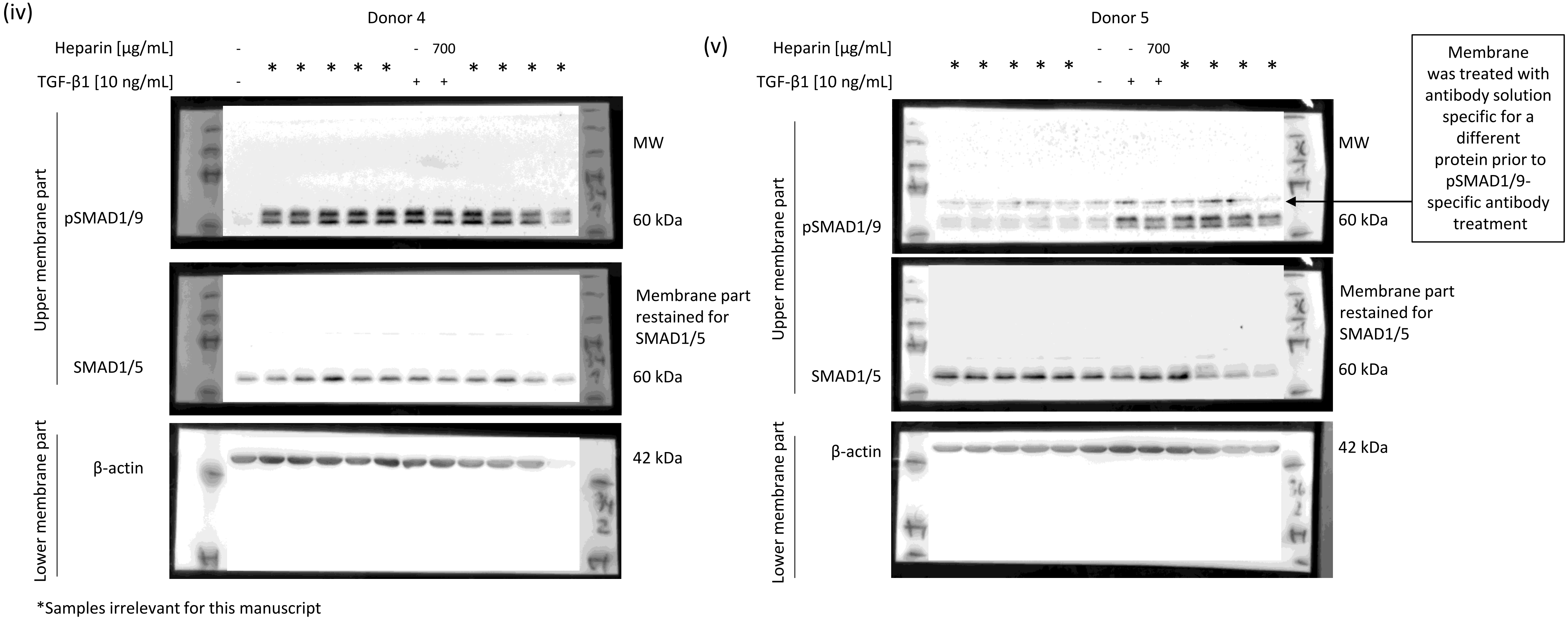

Supplementary Material S1. Western blots included in this study shown as full uncropped images.

F (i)

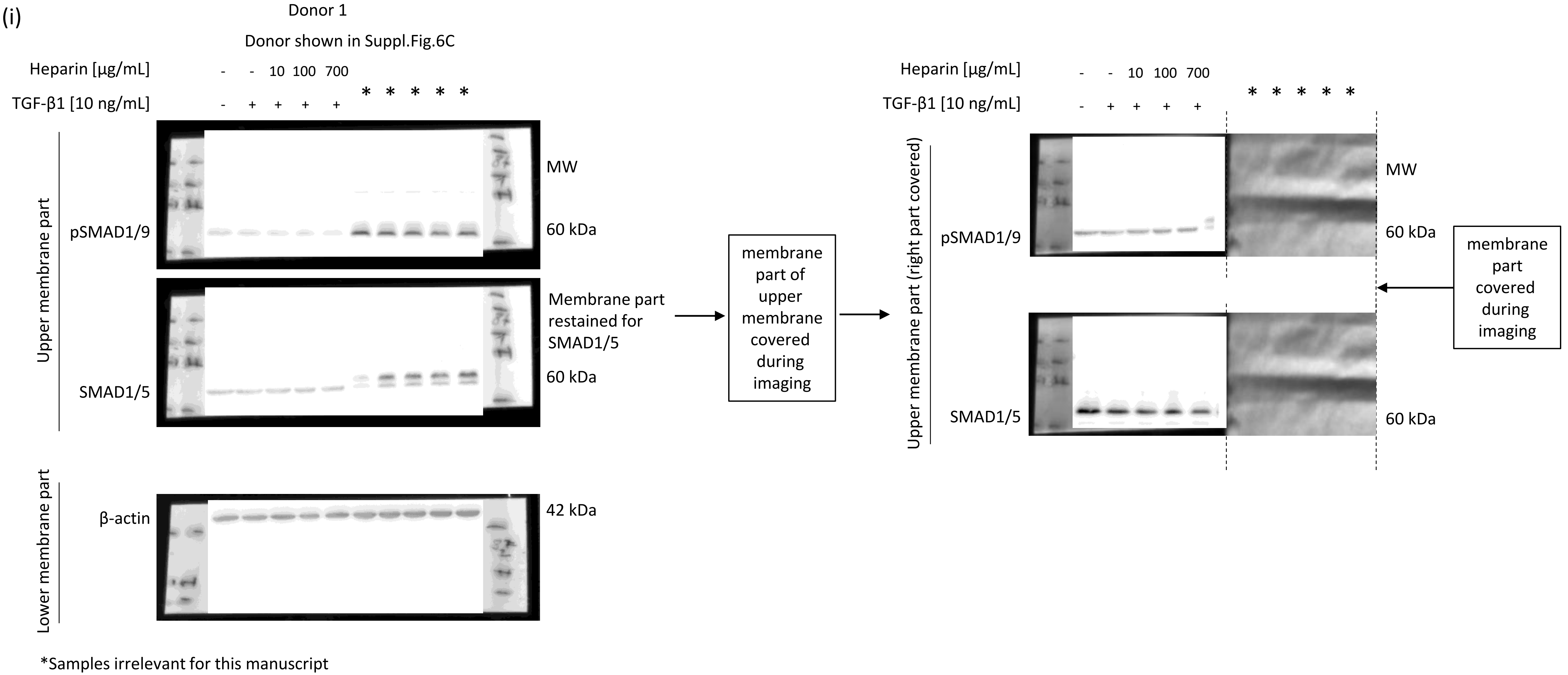

Supplementary Material S1. Western blots included in this study shown as full uncropped images.

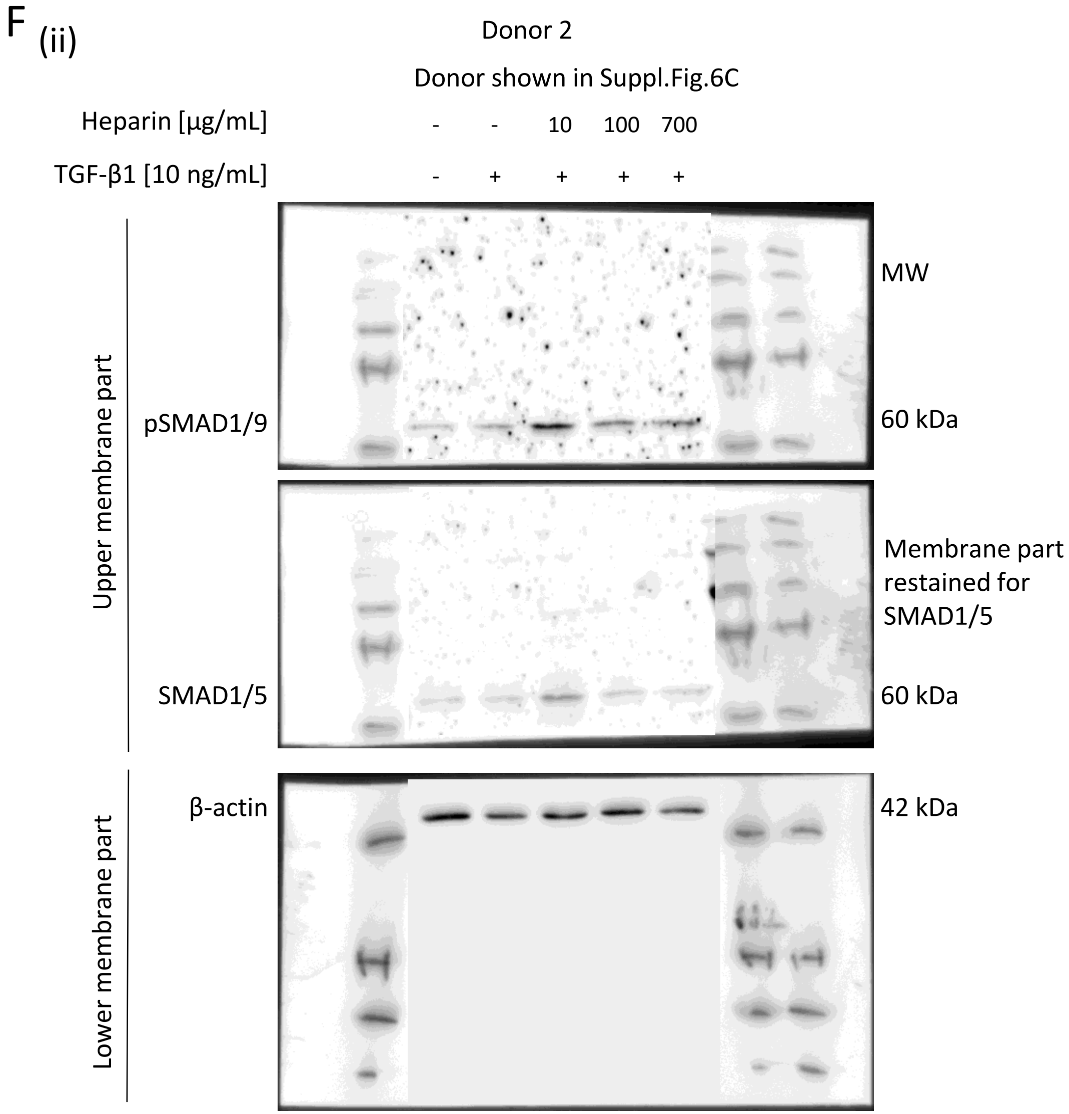

Supplementary Material S1. Western blots included in this study shown as full uncropped images.

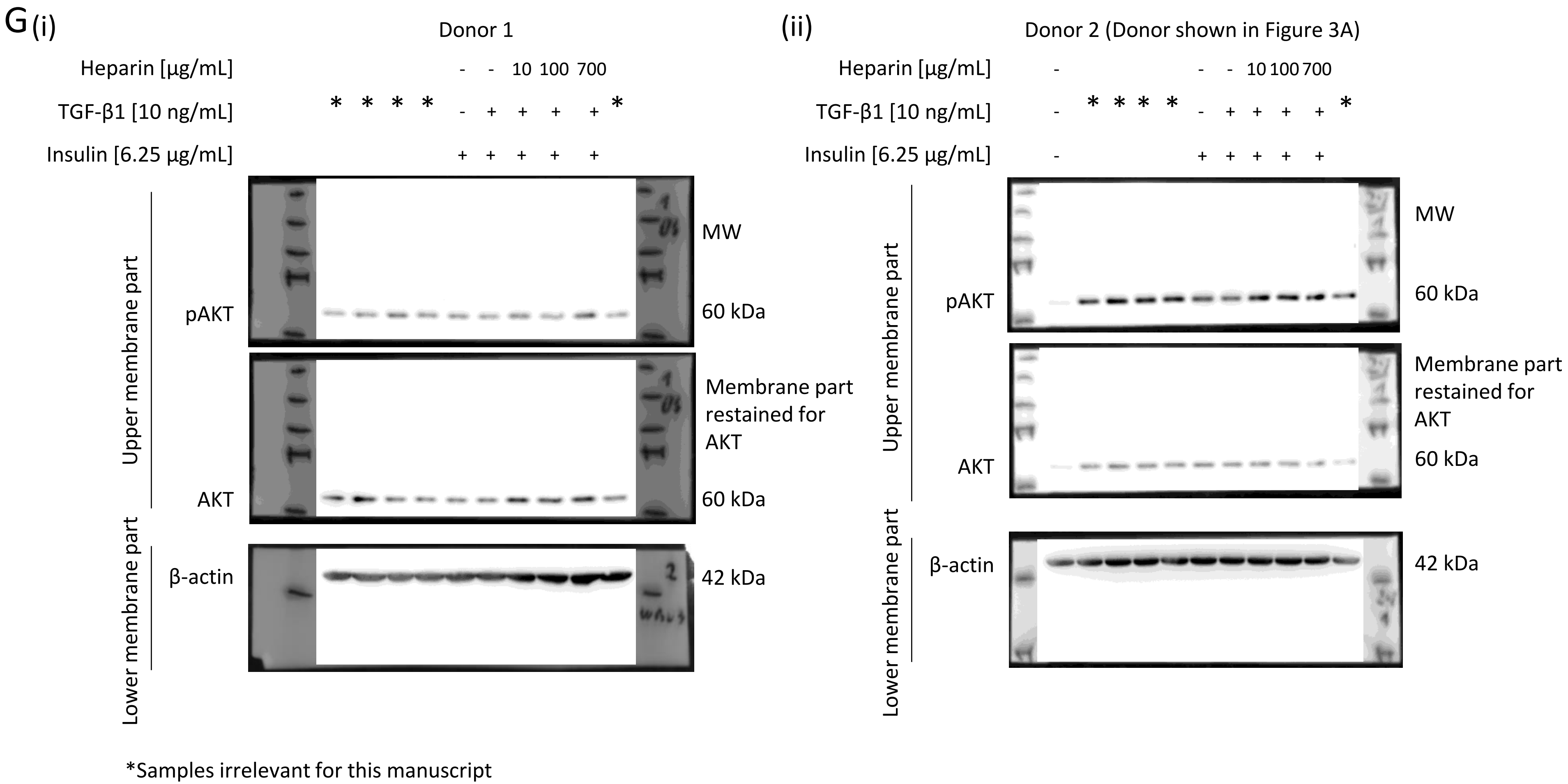

Supplementary Material S1. Western blots included in this study shown as full uncropped images.

(iii)

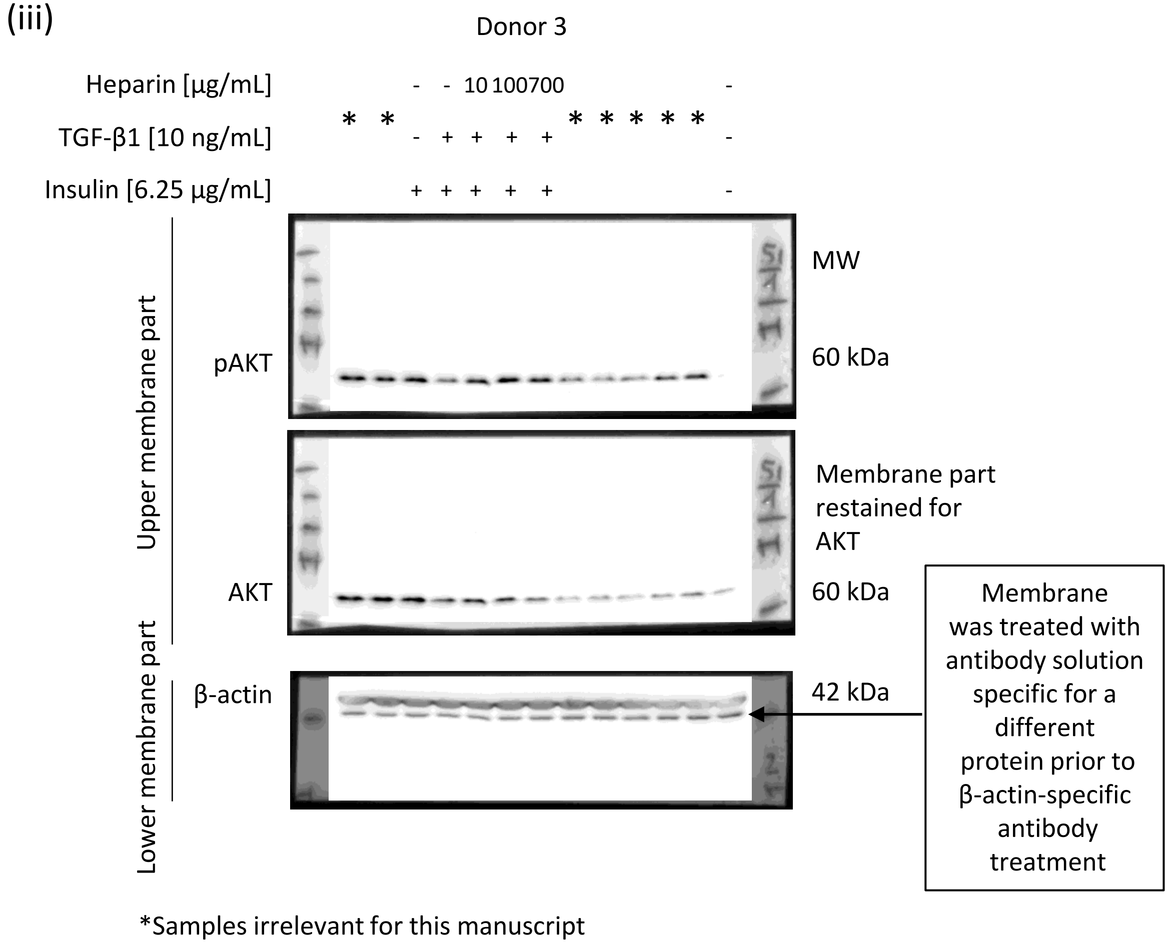

Supplementary Material S1. Western blots included in this study shown as full uncropped images.

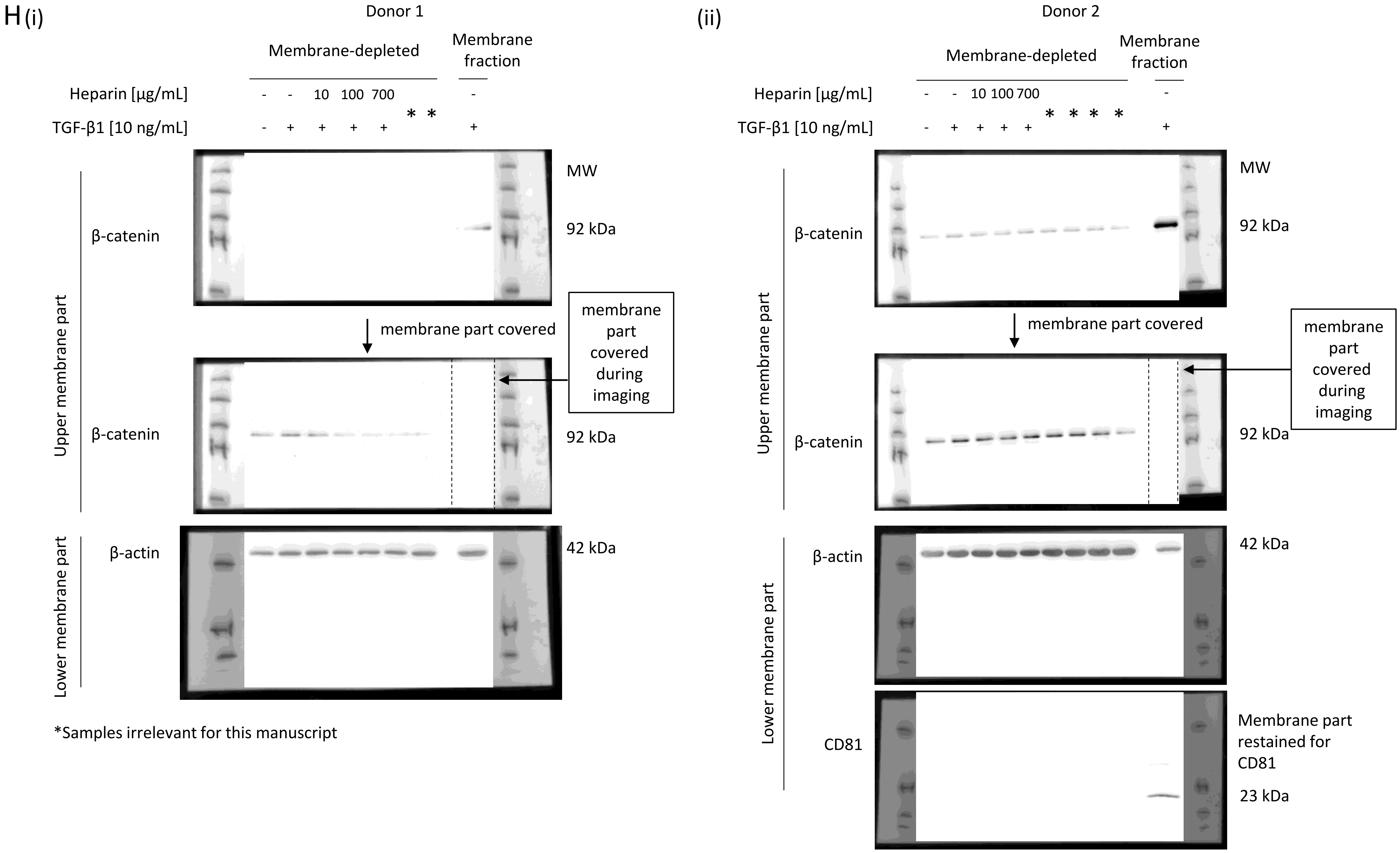

Supplementary Material S1. Western blots included in this study shown as full uncropped images.

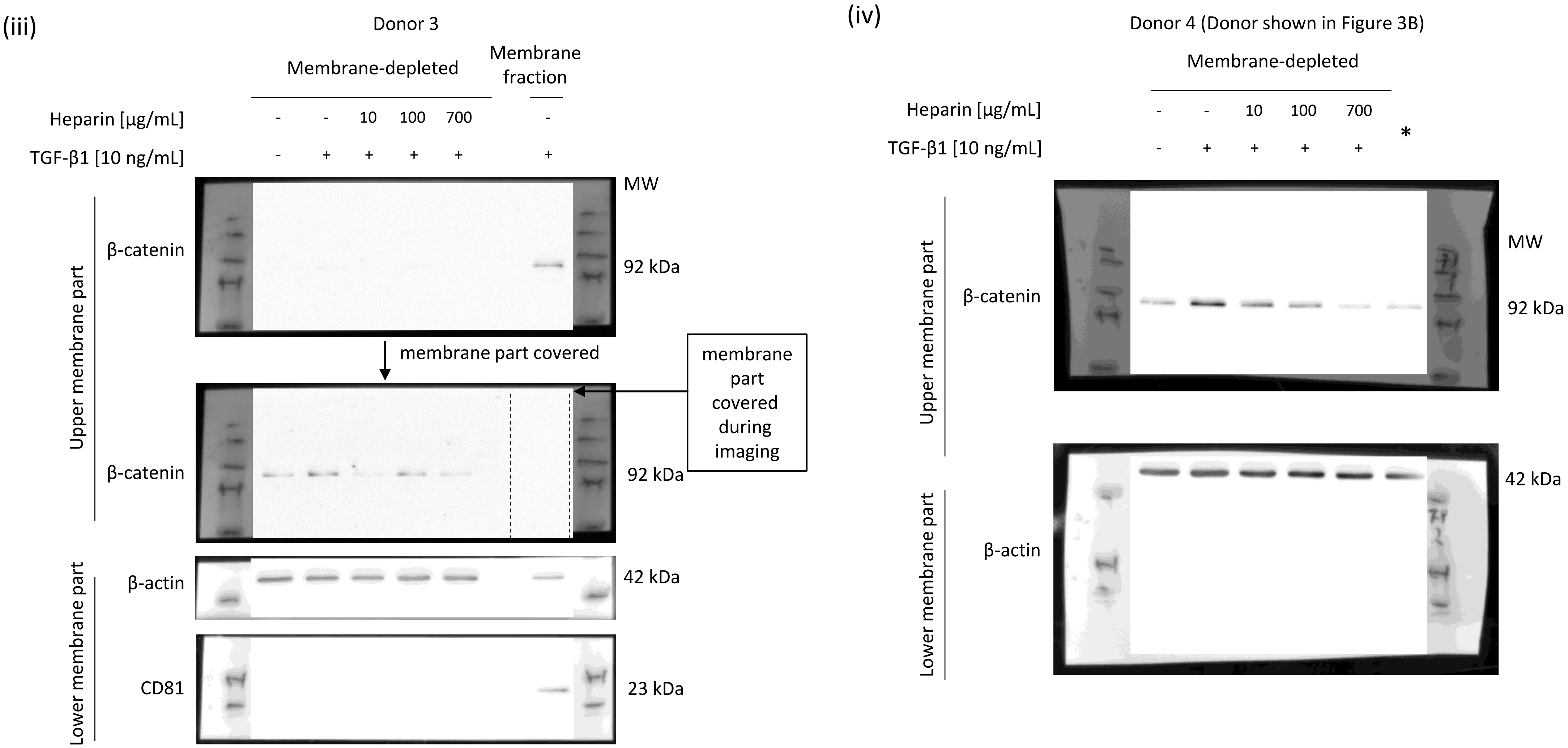

*Samples irrelevant for this manuscript

Supplementary Material S1. Western blots included in this study shown as full uncropped images.

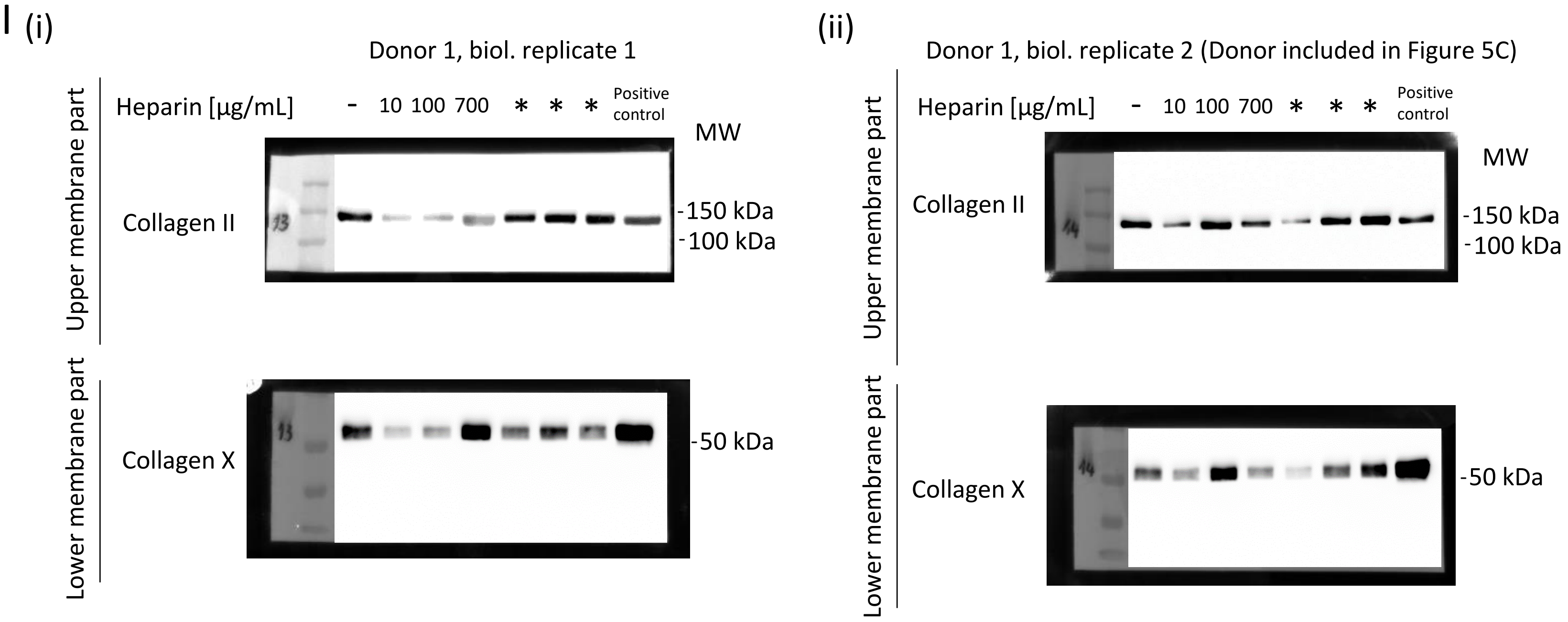

*Samples irrelevant for this manuscript

Supplementary Material S1. Western blots included in this study shown as full uncropped images.

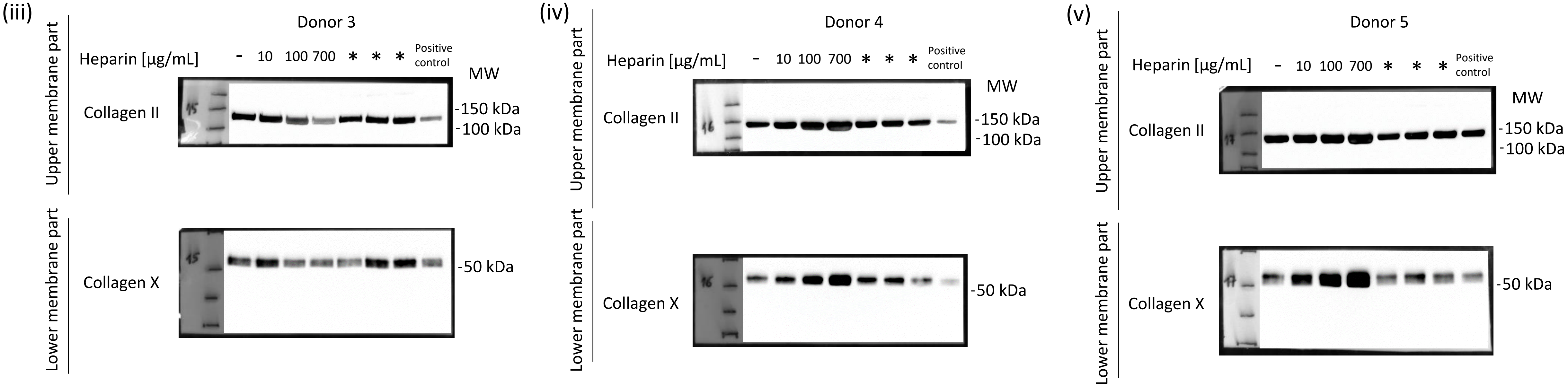

*Samples irrelevant for this manuscript

Supplementary Material S1. Western blots included in this study shown as full uncropped images.
