# Supplementary material for: Dichotomous SMAD2/3 regulation and selective antihypertrophic activity of heparin during in vitro chondrogenesis of mesenchymal stromal cells
Source: Cell Mol Biol Lett. 2026 Mar 17;31:51. doi: 10.1186/s11658-026-00899-8 (PMC13064404; doi:10.1186/s11658-026-00899-8)
Supplement: Supplementary file 12 — Additional file 12 (Supplementary Table S1. Alphabetical list of primer sequences utilized for qPCR analysis). [file 11658_2026_899_MOESM12_ESM.pdf]

Supplementary Table S1: Alphabetical list of primer sequences utilized for qPCR analysis.

Gen	Forward	Reverse
ALK2	5´-AAGGCAGGTATGGTGAGGT-3´	5´-AGAGTAGTAAGCTGAAGATAG-3´
ALK4	5´-CACGTGTGAGACAGATGGG-3´	5´-GGCGGTTGTGATAGACACG-3´
ALK5	5´-ATTACCAACTGCCTTATTATGA-3´	5´-CATTACTCTCAAGGCTTCAC-3´
ALPL	5´-CACCAACGTGGCTAAGAATG-3´	5´-TCAGCTGGATGGCCACATC-3´
ACAN	5´-GGAACCACTTGGGTCACG-3´	5´-GCACATGCCTTCTGCTT-3´
COL10A1	5´-TTTACGCTGAACGATACCAAA-3´	5´-TTGCTCTCCTCTTACTGCTAT-3´
COL2A1	5´-TGGCCTGAGACAGCATGAC-3´	5´-AGTGTTGGGAGCCAGATTGT-3´
COMP1	5´-CACTGACCTAGACGGCTTC-3´	5´-ATCAAAGTCGTCCTGGCACA-3´
CPSF6	5´-AAGATTGCCTTCATGGAATTGAG-3´	5´-TCGTGATCTACTATGGTCCCTCTCT-3´
GLI1	5´-TGCAGTAAAGCCTTCAGCAATG-3´	5´-TTTTCGCAGCGAGCTAGGAT-3´
HPRT	5´-AAGGGTGTTTATTCCTCATGGA-3´	5´-CCTCCCATCTCCTTCATCAC-3´
IBSP	5´-CAGGGCAGTAGTGACTCATCC-3´	5´-TCGATTCTTCATTGTTTTCTCCT-3´
IHH	5´-CGACCGCAATAAGTATGGAC-3´	5´-GGTGAGCGGGTGTGAGTG-3´
MEF2C	5´-GTATGGCAATCCCCGAAACT-3´	5´-ATCGTATTCTTGCTGCCTGG -3´
PTH1R	5´-GGTGAGGTGGTGGCTGT-3´	5´-AGCATGAAGGACAGGAAC-3´
SOX9	5´-GTACCCGCACTTGCACAAC-3´	5´-TCGCTCTCGTTCAGAAGTCTC-3´
THBS1	5´-GGCCTTTCTGTGAAAGTTGTA-3´	5´-AGACGCCATCTGTAGGCG-3´
